# Current-Induced Transistor Sensorics with Electrogenic Cells

**DOI:** 10.3390/bios6020018

**Published:** 2016-04-25

**Authors:** Peter Fromherz

**Affiliations:** Max-Planck-Institute for Biochemistry, Am Klopferspitz 18, Martinsried-München 82152, Germany; fromherz@biochem.mpg.de

**Keywords:** transistor, extracellular recording, ion channel, neuron, action potential

## Abstract

The concepts of transistor recording of electroactive cells are considered, when the response is determined by a current-induced voltage in the electrolyte due to cellular activity. The relationship to traditional transistor recording, with an interface-induced response due to interactions with the open gate oxide, is addressed. For the geometry of a cell-substrate junction, the theory of a planar core-coat conductor is described with a one-compartment approximation. The fast electrical relaxation of the junction and the slow change of ion concentrations are pointed out. On that basis, various recording situations are considered and documented by experiments. For voltage-gated ion channels under voltage clamp, the effects of a changing extracellular ion concentration and the enhancement/depletion of ion conductances in the adherent membrane are addressed. Inhomogeneous ion conductances are crucial for transistor recording of neuronal action potentials. For a propagating action potential, the effects of an axon-substrate junction and the surrounding volume conductor are distinguished. Finally, a receptor-transistor-sensor is described, where the inhomogeneity of a ligand–activated ion conductance is achieved by diffusion of the agonist and inactivation of the conductance. Problems with regard to a development of reliable biosensors are mentioned.

## 1. Introduction

**Figure 1:**
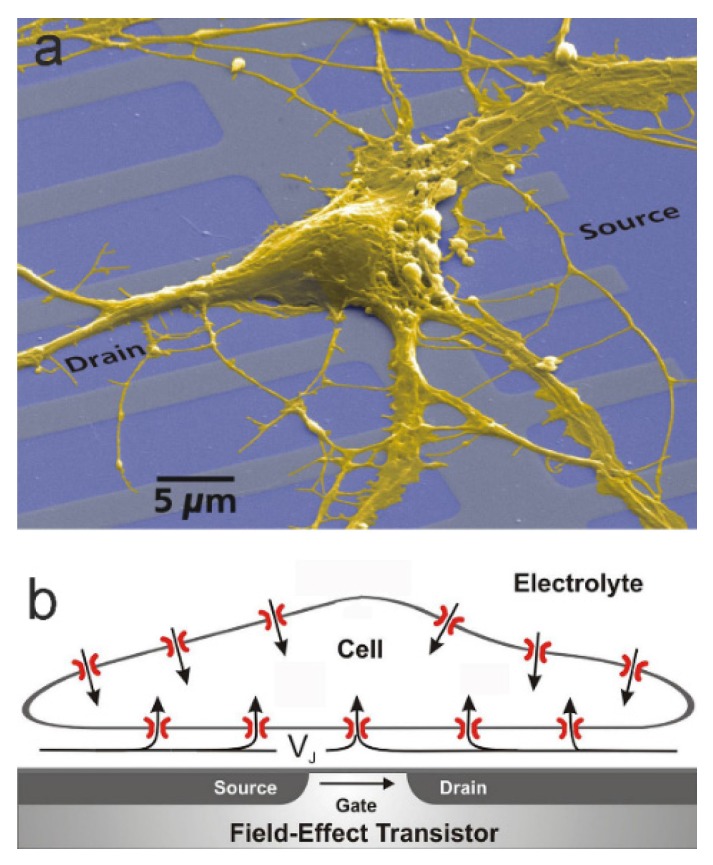
Planar cell-substrate junction with an EOSFET (electrolyte-oxide-semiconductor field-effect transistor), implementing the coupling of ionic and electronic currents: (**a**) Electronmicrograph of a cultured nerve cell from rat hippocampus on the silica surface of a silicon chip with an array of EOSFETs (by permission [3]); (**b**) Schematic cross section (not to scale). A cleft of about 50 *nm* with extracellular electrolyte separates the cell membrane and the silica surface. Ionic and capacitive currents through the cell membrane give rise to a current flow along the cleft. The ionic current induces a voltage *V_J_* with respect to the bath and modulates the electronic source-drain current at a constant gate voltage applied to a reference electrode in the bath.

The electrolyte-oxide-semiconductor field-effect transistor (EOSFET) has been introduced as an ion sensitive sensor (ISFET), where the interaction of ions with the gate oxide induces a changed source-drain current at a constant gate voltage applied to a reference electrode in the bath electrolyte [1]. A more specific interface-induced response can be achieved for biological analytes when the gate oxide is modified by suitable coatings [2]. However, response of an EOSFET may also be induced by ionic currents in the electrolyte, which give rise to a changed electrical potential beyond the electrical double layer of the oxide. The present paper describes the concepts of current-induced transistor sensorics for electroactive cells with a focus on planar cell-substrate junctions. The situation is illustrated in Figure 1 [3]: the adherent cell membrane and the semiconductor chip are separated by a thin cleft of extracellular electrolyte. Ionic and capacitive currents through the membrane give rise to a current flow along the cleft. That ionic current induces a voltage $V_{J}$ with respect to the bath and modulates the electronic source-drain current of a transistor at constant gate voltage.

Three issues are addressed in particular: (i) the cell-substrate junction is described as a planar capacitive core-coat conductor, which allows relating the cellular currents with changes of the electrical potential and changes of ion concentrations in the junction; (ii) in certain situations, the current-induced response is superposed by the interface-induced response of an ion-sensitive gate due to changed ion concentrations; and (iii) the inhomogeneous distribution of ion conductances in the cell membrane is crucial for a current-induced transistor response with intact electrogenic cells.

This paper is a scholarly overview of concepts and does not intend to provide a comprehensive review of the field, though some inconsistencies in the literature are addressed. The examples for various kinds of transistor recordings with electroactive cells are chosen from studies in my own lab.

The structure of the paper is as follows: First, the physics of the EOSFET is considered in order to identify the current-induced and the interface-induced transistor response with electrogenic cells. Then, the theory of a planar capacitive core-coat conductor is presented, which relates cellular currents with changes of the electrical potential and of ion concentrations in a cell-substrate junction. The dynamics of a core-coat conductor is described, and the reduction to a one-compartment model is derived for constant and for changing ion concentrations. On that basis, the transistor recording of voltage-activated ion channels is considered for an intracellular voltage-clamp. In this context, the role of an ion-sensitive gate oxide and the issue of inhomogeneous membrane conductances are pointed out. Current-induced transistor sensorics with intact cells is described for neuronal action potentials recorded at soma and axon, and for a receptor-cell-transistor biosensor.

Several technical issues have to be envisaged for cell-based transistor sensorics: (i) The gate must be smaller than a cell, and far smaller than a traditional transistor sensorics gate. As a consequence, the noise power, which is reciprocal to the gate area, becomes a problem; (ii) Current-induced extracellular voltages are in the range of a few microvolts to a few millivolts, lower than usual interface-induced signals. The signal-to-noise ratio is a critical issue; (iii) The transistor signals vary due to a variability of the cells, to the variable interactions of cells and substrate, and to a variable position of the transistor in the cell-substrate junction. These problems are not addressed here.

## 2. Transistor Sensorics with Cellular Currents

First some general aspects of the EOSFET are considered to avoid misunderstandings. On that basis, the interface-induced and current-induced responses of transistor recording are identified for electrogenic cells.

*Gate voltage and electrical potential*. The source–drain current of an EOSFET is determined by band bending, the drop of the electrical potential in the semiconductor $\Delta \varphi_{Si}^{S}=\varphi_{Si}^{S}-\varphi_{Si}^{\infty }$ from its surface at the gate oxide to the bulk [4]. Band bending is controlled by a gate voltage $V_{G}^{EOS}$ that is applied between the wires joined to a reference electrode (usually Ag/AgCl) in the bulk electrolyte and to the backside metal contact of the bulk semiconductor. (Practically, a voltage $V_{Si}=-V_{G}^{EOS}$ is applied to the semiconductor with the electrode on ground potential.) We consider the relation of $V_{G}^{EOS}$ with the difference $\varphi_{E}^{\infty }-\varphi_{Si}^{\infty }$ of the electrical potentials in the bulk electrolyte and in the bulk semiconductor, and then the relation of that difference with the band bending $\Delta \varphi_{Si}^{S}$.

The metal wires (identical materials) are in electronical equilibrium with the metal contacts (different materials) of the electrolyte and of the semiconductor, respectively, and these contacts are in electronical equilibrium with the redox solution of the reference electrode and with the bulk semiconductor, respectively. These equilibrations are described as alignments of the electrochemical potential of electrons (Fermi energies) ${\tilde{\mu }}_{k}^{el}=\mu_{k}^{el}-e_{0}\varphi_{k}$ ($e_{0}$ elementary charge) in the different phases with components of an electronic chemical potential and the electrical potential. (For the quasi-Fermi energy of a redox solution see [5].) As a consequence, the gate voltage controls the difference ${\tilde{\mu }}_{red/ox}^{el}-{\tilde{\mu }}_{Si}^{el}$ of the electronic electrochemical potentials in the redox solution and in the semiconductor [6]. When we take into account the electrical equilibration of the redox solution and the bulk electrolyte through a salt-bridge with $\varphi_{red/ox}^{}=\varphi_{E}^{\infty }$, the relation between the gate voltage and the difference of the electrical potentials in the bulk electrolyte and the bulk semiconductor is expressed by Equation (1).

(1) $$-e_{0}V_{G}^{EOS}=\left(\mu_{red/ox}^{el}-\mu_{Si}^{el}\right)-e_{0}\left(\varphi_{E}^{\infty }-\varphi_{Si}^{\infty }\right)$$

The electronic chemical potentials in the redox solution and in the semiconductor are important for the value of the flatband and the threshold voltage of an EOSFET (see Appendix A), but irrelevant for changes of the gate voltage around a working voltage of the field-effect transistor above the threshold of strong inversion with $\delta V_{G}^{EOS}=\delta \left(\varphi_{E}^{\infty }-\varphi_{Si}^{\infty }\right)$.

The overall difference $\varphi_{E}^{\infty }-\varphi_{Si}^{\infty }$ of the electrical potentials can be partitioned into several components according to Equation (2a) with the potential drops $\Delta \varphi_{E}^{S}$ and $\Delta \varphi_{Si}^{S}$ in the electrolyte and in the semiconductor (both from the surface at the oxide to the bulk), with the potential jumps $\Delta \varphi_{OxE}^{}$ and $\Delta \varphi_{OxSi}^{}$ across the interfaces oxide-to-electrolyte and oxide-to-semiconductor, and with the potential drop $\Delta \varphi_{Ox}$ across the oxide (electrolyte-to-semiconductor). The latter is split into contributions of a fixed charge $\sigma_{OxSi}$ at the oxide/semiconductor interface and of the space charge in the semiconductor according to Equation (2b) for a p-channel transistor in depletion approximation (gate capacitance $c_{Ox}$, permittivity $\varepsilon_{Si}\varepsilon_{0}$, donor concentration $N_{D}$) [4].

(2a) $$\varphi_{E}^{\infty }-\varphi_{Si}^{\infty }=-\Delta \varphi_{E}^{S}-\Delta \varphi_{OxE}^{}+\Delta \varphi_{Ox}+\Delta \varphi_{OxSi}^{}+\Delta \varphi_{Si}^{S}$$

(2b) $$\Delta \varphi_{Ox}=-c_{Ox}^{-1}\left[\sigma_{OxSi}+\sqrt{2\varepsilon_{Si}\varepsilon_{0}e_{0}N_{D}\left(-\Delta \varphi_{Si}^{S}\right)}\right]$$

The fabrication of a chip determines the potential drop $-\sigma_{OxSi}/c_{Ox}$ as well as the potential jump $\Delta \varphi_{OxSi}^{}$. The conditions of the electrolyte determine the potential jump $\Delta \varphi_{OxE}^{}$ (Si=O and Si-OH groups, oriented H_2_O, double layer of Si-O^−^ groups and adsorbed ions) [7]. The term quasi-Helmholtz layer is used in analogy to a metal/electrolyte interface with $\Delta \varphi_{OxE}^{}\equiv \Delta \varphi_{Hh}$. The conditions of the electrolyte also determine the potential drop $\Delta \varphi_{E}^{S}$ with a component $\Delta \varphi_{GCh}$ in a diffuse electrical double layer (Gouy-Chapman) and a component $\Delta \varphi_{E}$ in the free electrolyte beyond the double layer, which is induced by a current flow in the electrolyte. $\Delta \varphi_{E}$ vanishes in equilibrium for the electroneutral electrolyte. Considering Equations (1) and (2a), a changed gate voltage $\delta V_{G}^{EOS}$ is partitioned into a changed current-induced drop of the electrical potential in the free electrolyte, a changed potential drop across the oxide/electrolyte interface (with Gouy-Chapman and quasi-Helmholtz layer), and a changed band bending (with the changed potential drop $\Delta \varphi_{Ox}^{spch}$ due the space charge) according to Equation (3).

(3) $$\delta V_{G}^{EOS}=-\delta \Delta \varphi_{E}-\delta \left(\Delta \varphi_{GCh}^{}+\Delta \varphi_{Hh}^{}\right)+\delta \left(\Delta \varphi_{spch}^{Ox}+\Delta \varphi_{S}^{Si}\right)$$

*Transistor and sensor.* Equation (3) can be read in two ways: (i) In a transistor mode, the conditions in the electrolyte are constant. A changed gate voltage $\delta V_{G}^{EOS}$ induces a changed band bending with a changed source-drain current above the threshold of strong inversion; (ii) In a sensor mode, the gate voltage is held constant. A changed electrical potential in the free electrolyte or across the Gouy-Chapman and quasi-Helmholtz layers gives rise to a changed band bending. The resulting change of the source-drain current can be expressed as an apparent change of the gate voltage with Equation (4).

(4) $$\delta V_{Gapp}^{EOS}=\delta \Delta \varphi_{E}+\delta \left(\Delta \varphi_{GCh}^{}+\Delta \varphi_{Hh}^{}\right)$$

The interface-induced response accounts for the classical ISFET sensorics, whereas the current-induced response is a typical issue of the transistor sensorics with electrogenic cells.

*Interpretation.* The goal of transistor sensorics with electrogenic cells is an interpretation of a signal $\delta V_{Gapp}^{EOS}$ in terms of the generating cellular currents. According to the core-coat conductor theory of a cell-substrate junction, as considered in the next section, cellular currents induce changes $\delta V_{J}=\delta \Delta \varphi_{E}$ of the electrical potential in the junction and changes $\delta c_{J}^{i}$ of the ion concentrations, which give rise to an interface-induced response $\delta \left(\Delta \varphi_{GCh}^{}+\Delta \varphi_{Hh}^{}\right)$.

The interpretation of a signal $\delta V_{Gapp}^{EOS}$ is involved if the interface-induced response plays a role. In a first step, the interface-induced response must be identified in the total transistor signal. This task is difficult because the peculiar (slow) dynamics of ion concentrations affects the interface-induced response as well as the current-induced response. In a second step, a calibration in terms of concentration changes $\delta c_{J}^{i}$ is required. In principle, such a relation is obtained by titrating a cell-free transistor with the ion concentration $c_{E}^{i}$ in the bath electrolyte yielding an ion sensitivity of the gate per decade $S_{GE}^{i}$ according to Equation (5) for a limited concentration range.

(5) $$\delta \left(\Delta \varphi_{GCh}^{}+\Delta \varphi_{Hh}^{}\right)=S_{GE}^{i}\;\delta \left(\log\;c_{E}^{i}\right)$$

However, the chemical nature of the gate surface is affected by cell adhesion. With an unknown ion sensitivity $S_{GJ}^{i}\neq S_{GE}^{i}$ in the junction, an assignment of a changing concentration $\delta c_{J}^{i}$ is not possible.

An unequivocal interpretation of transistor signals with electroactive cells is achieved if the interface–induced response plays no role with $\delta V_{Gapp}^{EOS}=\delta \Delta \varphi_{E}$. Two situations can be envisaged: (i) the activation of cellular ion currents is so short that changes of the ion concentrations in the junction can be neglected; and (ii) the gate is chemically passivated such that changing ion concentrations are not able to induce an interface-induced response.

Finally, it may be noted that the sensor concept as sketched here for the EOSFET is also applicable for an electrolyte-oxide-metal-oxide-semiconductor (EOMOS) transistor and – *mutatis mutandis* – for other potentiometric electrodes (see Appendix B).

## 3. Planar Capacitive Core-Coat Conductor

Ionic and capacitive currents through an adherent cell membrane induce changes of the electrical potential and of the ion concentrations in a cell-substrate junction. These relations are described in terms of a planar capacitive core-coat conductor.

The thin film of extracellular electrolyte in a cell-substrate junction is insulated from the intracellular electrolyte by the plasma membrane and from the semiconductor by an oxide layer (Figure 1b). For cultured mammalian cells, the distance of the two insulating coats is $d_{J}\approx 50\;nm$ as measured by fluorescence interference contrast (FLIC) microscopy [8]. It is far larger than the Debye length of the electrolyte with $l_{Debye}\approx 1\;nm$ and far smaller than the width of the adhesion area with a radius in the range of $a_{J}\approx 10\;\mu m$. The condition $l_{Debye}<<d_{J}<<a_{J}$ is similar to a relation for neuronal axons, where a thin thread of intracellular electrolyte is insulated from the bath by the cylindrical plasma membrane with $l_{Debye}<<a_{M}<<l_{axon}$ for Debye length, radius, and length.

The electrical features of an axon are characterized by three properties: (i) the cytoplasmatic core as well as the bath are electroneutral beyond thin space charge layers at the membrane; (ii) the electrical charge in these space charge layers determines the voltage across the membrane by a capacitor relation; and (iii) the profiles of the ion concentrations and of the electrical potential across the narrow core are neglected as compared with the profiles along the core. As a consequence, axons are described as 1D linear capacitive core-coat conductors with ionic currents through the coat and along the core, usually for constant intracellular ion concentration [9] but occasionally also for changing ion concentrations [10]. With the same rationale, cell-substrate junctions have been described as 2D planar capacitive core-coat conductors for constant [11] and for changing ion concentrations [12,13].

*Balance of ion flux.* The ion flux through the adherent membrane in a cell-substrate junction gives rise to a changing (in time) ion concentration in the core and to a changing (in space) ion flux along the core. The flux along the core is driven by gradients (in two dimensions) of the electrical potential $\varphi_{J}$ (drift mobility $u_{J}^{i}$) and of the concentration $c_{J}^{i}$ (diffusion coefficient $D_{J}^{i}=k_{B}Tu_{J}^{i}$, thermal energy $k_{B}T$). The flux balance is described by Equation (6) with the ionic charge number $z_{i}$ and the electrical current $i_{JM}^{i}$ per unit area of the adherent membrane [14].

(6) $$-\nabla \left[d_{J}z_{i}^{2}e_{0}^{2}u_{J}^{i}\left(c_{J}^{i}\nabla \varphi_{J}+\frac{k_{B}T}{z_{i}e_{0}}\nabla c_{J}^{i}\right)\right]+d_{J}z_{i}e_{0}\frac{\partial c_{J}^{i}}{\partial t}=i_{JM}^{i}$$

*Balance of charge.* Changing ion concentrations give rise to a changing electrical charge in the junction. A net charge $\sum d_{J}z_{i}e_{0}c_{J}^{i}\;\neq 0$ in the core electrolyte would violate the electroneutrality condition. That charge is located in thin space charge layers along membrane and semiconductor oxide, respectively, with counter-charges induced across the two coats [15]. A changing charge is related with changing differences $\varphi_{J}-\varphi_{cell}^{\infty }$ and $\varphi_{J}-\varphi_{Si}^{\infty }$ of the electrical potential in the junction with respect to cell and semiconductor by a differential capacitor relation. With a capacitance $c_{M}$ of the membrane and an average capacitance $c_{S}$ of the substrate (differing from the local capacitance $c_{Ox}$ of the gate oxide), we obtain Equation (7). (A different approach has been chosen in [16], where the net charge is assigned to a space charge in the electrolyte film. See Appendix C).

(7) $$\sum d_{J}z_{i}e_{0}\frac{\partial c_{J}^{i}}{\partial t}\;=c_{M}\frac{\partial \left(\varphi_{J}-\varphi_{cell}^{\infty }\right)}{\partial t}+c_{S}\frac{\partial \left(\varphi_{J}-\varphi_{Si}^{\infty }\right)}{\partial t}$$

*Current balance and limit of constant ion concentrations.* The balance of electrical charge (Equation (7)) can be combined with the dynamics of the ion concentrations (Equation (6)). We obtain Equation (8) for the balance of the total current, when the electrical potentials in junction, cell, and semiconductor are expressed as voltages with respect to the bulk electrolyte with $V_{J}=\varphi_{J}^{}-\varphi_{E}^{\infty }$, $V_{M}=\varphi_{cell}^{\infty }-\varphi_{E}^{\infty }$, and $V_{Si}=\varphi_{Si}^{\infty }-\varphi_{E}^{\infty }$ defining a junction capacitance $c_{J}=c_{M}+c_{S}$ (Distinguish capacitance $c_{J}$ and ion concentrations $c_{J}^{i}$!).

(8) $$-\sum \nabla \left[d_{J}z_{i}^{2}e_{0}^{2}u_{J}^{i}\left(c_{J}^{i}\nabla V_{J}+\frac{k_{B}T}{z_{i}^{}e_{0}^{}}\nabla c_{J}^{i}\right)\right]+c_{J}\frac{\partial V_{J}}{\partial t}=c_{S}\frac{dV_{Si}}{dt}+c_{M}\frac{dV_{M}}{dt}+\sum i_{JM}^{i}$$

For small changes of the ion concentrations, the diffusion term in Equation (8) can be cancelled and the concentrations can be excluded from the spatial derivative of the drift term. With a conductivity $1/\rho_{J}=\sum z_{i}^{2}e_{0}^{2}u_{J}^{i}c_{J}^{i}$ of the electrolyte in the junction, we obtain Equation (9).

(9) $$-\nabla \left(\frac{d_{J}}{\rho_{J}}\nabla V_{J}\right)+c_{J}\frac{\partial V_{J}}{\partial t}=c_{S}\frac{dV_{Si}}{dt}+c_{M}\frac{dV_{M}}{dt}+\sum i_{JM}^{i}$$

This “2D cable equation” for a planar capacitive core-coat conductor with constant ion concentrations is homologous to the common 1D cable equation for neuronal axons. Two peculiarities, however, must be noted: (i) the voltage in the junction $V_{J}$ depends on the voltage changes in the two different adjacent media of cell and substrate; and (ii) at its periphery, the core conductor is open to the bath electrolyte.

*Transistor response*. The capacitive and ionic currents through the adherent membrane of a cell-substrate junction give rise to a coupled dynamics of the extracellular ion concentrations $c_{J}^{i}$ and of the extracellular electrical potential $\varphi_{J}$, as described by Equations (6) and (7). The apparent change of the gate voltage $\delta V_{Gapp}^{EOS}$ for an EOSFET in the junction (Equation (4)) is caused by a current-induced response due to a changing voltage in the junction and by an interface-induced response due to changing ion concentrations according to Equation (10).

(10a) $$\delta \;\Delta \varphi_{E}=\delta V_{J}$$

(10b) $$\delta \left(\Delta \varphi_{GCh}^{}+\Delta \varphi_{Hh}^{}\right)=\sum S_{GJ}^{i}\;\delta \left(\log\;c_{J}^{i}\right)$$

## 4. Dynamics of Planar Capacitive Core-Coat Conductor

The time constants for changes of the voltage $V_{J}$ and of the ion concentrations $c_{J}^{i}$ in a cell-substrate junction are crucial for the response of a transistor to cellular currents. We consider a circular junction (radius $a_{J}$, area $A_{J}=a_{J}^{2}\pi$) with a homogeneous sheet resistance $r_{J}=\rho_{J}/d_{J}$.

*Voltage dynamics.* The dynamics of the junction voltage $V_{J}$ for constant ion concentrations is described by Equation (11a) with a radial coordinate a (at a constant bias voltage of the semiconductor). The ionic membrane currents depend on the conductances per unit area $g_{JM}^{i}$ according to Equation (11b) , which are driven by the transmembrane voltage and the reversal voltage $V_{M0}^{i}$ (determined by the difference of the logarithmic concentrations across the membrane).

(11a) $$-\frac{1}{r_{J}}\left(\frac{\partial^{2}}{\partial a^{2}}+\frac{1}{a}\frac{\partial }{\partial a}\right)V_{J}+c_{J}\frac{\partial V_{J}}{\partial t}=c_{M}\frac{dV_{M}}{dt}+\sum i_{JM}^{i}$$

(11b) $$i_{JM}^{i}=g_{JM}^{i}\left(V_{M}-V_{J}-V_{M0}^{i}\right)$$

First, we apply a voltage step $\Delta V_{M}^{0}$ to a cell without membrane conductances for a boundary condition $V_{J}\left(a_{J}\right)=0$ and an initial condition $V_{J}\left(t=0\right)=0$. The sharp pulse of capacitive current induces a junction voltage $\Delta V_{M}^{0}c_{M}/c_{J}$, which decays according to Equation (12) with a relaxation of modes (Bessel functions $J_{0}$, $J_{1}$, zeros $z_{0,n}\;(n=1,2,..)$ of $J_{0}$) [17] (p. 199).

(12) $$V_{J}\left(a,t\right)=\Delta V_{M}^{0}\frac{\;c_{M}}{c_{J}}\sum_{n=1}^{\infty }\frac{2}{z_{0,n}}\frac{J_{0}\left(z_{0,n}a/a_{J}\right)}{\;J_{1}\left(z_{0,n}\right)}\exp\left(-\frac{z_{0,n}^{2}}{r_{J}c_{J}a_{J}^{2}}t\right)$$

To reveal the response to an ion current, we apply a voltage step to a cell with a single ion conductance $g_{JM}^{i}$ starting from $V_{M}=V_{M0}^{i}$. Besides the capacitive response, an increasing junction voltage is induced according to Equation (13) for $\left|V_{J}\right|<<\left|V_{M}-V_{M0}^{i}\right|$ with a stationary parabolic profile [17] (p. 204).

(13) $$V_{J}\left(a,t\right)=\Delta V_{M}^{0}g_{JM}^{i}\frac{r_{J}A_{J}}{4\pi }\left[\left(1-\frac{a^{2}}{a_{J}^{2}}\right)-\sum_{n=1}^{\infty }\frac{8}{z_{0,n}^{3}}\frac{J_{0}\left(z_{0,n}a/a_{J}\right)}{\;J_{1}\left(z_{0,n}\right)}\exp\left(-\frac{z_{0,n}^{2}}{r_{J}c_{J}a_{J}^{2}}t\right)\right]$$

The complete response is described by a superposition of the Equations (12) and (13). The dynamics is characterized by the time constant $\tau_{J}^{\left(1\right)}=r_{J}c_{J}A_{J}/5.78\pi$ of the lowest mode.

**Figure 2:**
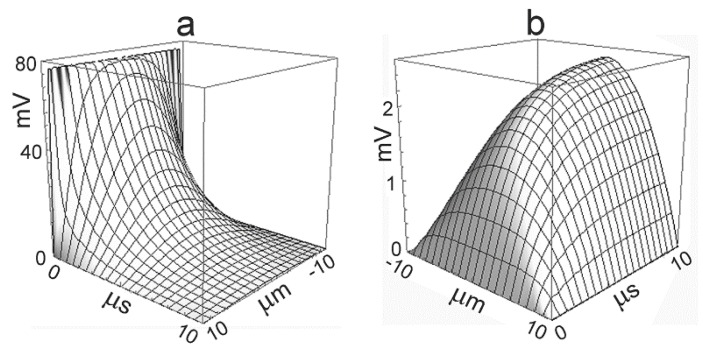
Time-dependent profiles of the extracellular voltage *V_J_(a,t)* in a circular cell-substrate junction upon intracellular stimulation by a voltage step of 100 *mV*: (**a**) response to capacitive current; and (**b**) response to ionic current. The dynamics in the microsecond range is determined by the time constant of the lowest mode of the core–coat conductor. For the parameters, see text. Note the different orientation and different voltage scales of the diagrams.

Typical parameters for cultured mammalian cells (HEK293, hippocampal neuron) are $a_{J}=10\;\mu m$, $r_{J}=10\;M\Omega$, $c_{M}=1\;\mu F/cm^{2}$, $c_{J}=1.3\;\mu F/cm^{2}$, and $g_{JM}^{i}\approx 10\;mS/cm^{2}$. The time-dependent profiles $V_{J}\left(a,t\right)$ upon a voltage step $\Delta V_{M}^{0}=100\;mV$ are plotted in Figure 2 for the capacitive current and for the ionic current [18]. The capacitive current induces an initial voltage of 77 *mV*. The ionic current gives rise to a stationary parabolic voltage profile with an amplitude of 2.5 *mV*. The dynamics is dominated by the time constant $\tau_{J}^{\left(1\right)}=2.25\;\mu s$.

*Quasi*-*stationary response.* The electrical time constant of a cell-substrate junction is in the microsecond range and far shorter than the time scale of the natural dynamics of capacitive and ionic currents of cells in the range of 0.1–1 *ms*. As a consequence, the junction voltage is almost relaxed upon stimulation. The 2D cable equation (Equation (9)) can be reduced to Equation (14) for a homogeneous junction and small voltages $\left|V_{J}\right|<<\left|V_{M}-V_{M0}^{i}\right|$.

(14) $$-\frac{d_{J}}{\rho_{J}}\nabla^{2}V_{J}=c_{M}\frac{dV_{M}}{dt}+\sum g_{JM}^{i}\left(V_{M}-V_{M0}^{i}\right)$$

In this quasi-stationary situation, the planar capacitive core-coat conductor appears as a sheet conductor that is driven from the cell by a 2D (capacitive and ionic) current-source density. There is a certain analogy to a volume conductor with a 3D current-source density [19,20]. Considering Equation (13), the quasi-stationary voltage profile is parabolic for a circular junction according to Equation (15).

(15) $$V_{J}\left(a,t\right)=\frac{r_{J}A_{J}}{4\pi }\left(1-\frac{a^{2}}{a_{J}^{2}}\right)\left[c_{M}\frac{dV_{M}}{dt}+\sum g_{JM}^{i}\left(V_{M}-V_{M0}^{i}\right)\right]$$

*Concentration dynamics.* The concentration dynamics of ions in a planar core-coat conductor is governed by drift and diffusion according to Equation (6). The drift term is determined by the electrical potential, which depends on the concentration dynamics of all ions (Equation (7)). When we disregard the drift, we obtain Equation (16) for the pure, independent ion diffusion in a homogeneous circular junction.

(16) $$-D_{J}^{i}\left(\frac{\partial^{2}}{\partial a^{2}}+\frac{1}{a}\frac{\partial }{\partial a}\right)c_{J}^{i}+\frac{\partial c_{J}^{i}}{\partial t}=\frac{i_{JM}^{i}}{d_{J}z_{i}e_{0}}$$

A step of ion current $\Delta \;i_{JM}^{i}$ induces a concentration change according to Equation (17) for a boundary condition $c_{J}^{i}\left(a_{J}\right)=c_{E}^{i}$ and an initial condition $c_{J}^{i}\left(t=0\right)=c_{E}^{i}$ with the concentration $c_{E}^{i}$ in the bulk electrolyte. The dynamics is characterized by the time constant $\tau_{J}^{\left(1\right)}=A_{J}/5.78\pi \;D_{J}^{i}$ of the lowest mode.

(17) $$c_{J}^{i}-c_{E}^{i}=\frac{\Delta i_{JM}^{i}}{z_{i}e_{0}\;d_{J}}\frac{A_{J}}{4\pi \;D_{J}^{i}}\left[\left(1-\frac{a^{2}}{a_{J}^{2}}\right)-\sum_{n=1}^{\infty }\frac{8}{z_{0,n}^{3}}\frac{J_{0}\left(z_{0,n}a/a_{J}\right)}{J_{1}\left(z_{0,n}\right)}\exp\left(-\frac{z_{0,n}^{2}D_{J}^{i}}{a_{J}^{2}}t\right)\right]$$

For $\Delta i_{JM}^{i}=1\;mA/cm^{2}$ (with $g_{JM}^{i}=10\;m\;S/cm^{2}$ and $V_{M}-V_{M0}^{i}=100\;mV$), $D_{J}^{i}=2\cdot 10^{-5}cm^{2}/s$, $d_{J}=70\;nm$, and $a_{J}=10\;\mu m$, a stationary concentration change up to 18.6 *mM* is attained with a time constant $\tau_{J,diff}^{\left(1\right)}=8.7\;ms$. The dynamics of ion concentrations is far slower than the dynamics of the voltage. It is illustrated by Figure 2b, if the time axis is rescaled by a factor $\tau_{J,diff}^{\left(1\right)}/\tau_{J}^{\left(1\right)}\approx 4000$. A significant concentration change appears only if the duration of the ion current is comparable with the time constant $\tau_{J,diff}^{\left(1\right)}$.

*Electrical characterization of cell-substrate junction*. The capacitive transient upon stimulation by a voltage-step (Equation (12), Figure 2a) can be used to determine the sheet resistance of a cell-substrate junction. More reliable, however, is the application of an ac voltage with an amplitude ${\underline{V}}_{M}\left(\omega \right)$ (angular frequency ω). The frequency-dependent voltage profile ${\underline{V}}_{J}\left(a,\omega \right)$ in a homogeneous circular junction without membrane conductance can be derived from Equation (11) according to Equation (18) with the modified Bessel function $I_{0}$. This approach has been used for leech neurons with an array of transistors in the cell–substrate junction [21].

(18) $${\underline{V}}_{J}\left(a,\omega \right)={\underline{V}}_{M}\frac{c_{M}}{c_{J}}\left[1-\frac{I_{0}\left(\gamma a\right)}{I_{0}\left(\gamma a_{J}\right)}\right],\gamma^{2}=i\omega \;r_{J}c_{J}$$

A related approach is the application of an ac voltage ${\underline{V}}_{Si}$ to the capacitor structure of a chip. The response ${\underline{V}}_{M}-{\underline{V}}_{J}$ across the adherent membrane has been probed with a voltage-sensitive fluorescent dye [22,23]. The relation of stimulus and response is similar to Equation (18) but more involved due to the coupled response of junction and cell. An average sheet resistance of $r_{J}=7.7M\Omega$ has been obtained for HEK293 cells on a silica surface coated with fibronectin and of $r_{J}=12M\Omega$ for hippocampal rat neurons on poly–lysine [24].

A particularly elegant method is the recording of the thermal voltage fluctuations in a cell-substrate junction [25]. The autocorrelation function of $V_{J}\left(t\right)$, averaged over a circular sensor with a radius $a_{S}$ in the center of a circular junction, is expressed by Equation (19) [26].

(19) $$\Phi_{}^{S}\left(\Delta t\right)=\frac{k_{B}T}{c_{J}a_{S}^{2}\pi }\sum_{n=1}^{\infty }\frac{4}{z_{0,n}^{2}}{\left[\frac{J_{1}\left(z_{0,n}a_{S}/a_{J}\right)}{J_{1}\left(z_{0,n}\right)}\right]}^{2}\exp\left(-\frac{z_{0,n}^{2}}{r_{J}c_{J}a_{J}^{2}}\Delta t\right)$$

Practically, the power spectral density (PSD) of the voltage fluctuations is probed, and the data are fitted with a theoretical PSD, which is obtained by Fourier transformation of the autocorrelation function. For low frequencies, it is given by Equation (20) [26].

(20) $$S_{f}^{S}=4k_{B}T\;r_{J}\left(\frac{1}{8\pi }+\frac{1}{2\pi }\ln\frac{a_{J}}{a_{S}}\right)$$

The PSD for rat neurons on poly–lysine has been probed with an EOSFET at low frequency with $S_{f}^{S}=4.7\cdot 10^{-14}V^{2}/Hz$ [25]. According to Equation (20), with $a_{S}/a_{J}=0.25$, a sheet resistance of $r_{J}=11M\Omega$ is obtained, which is similar to the resistance evaluated from the ac stimulation.

**Figure 3:**
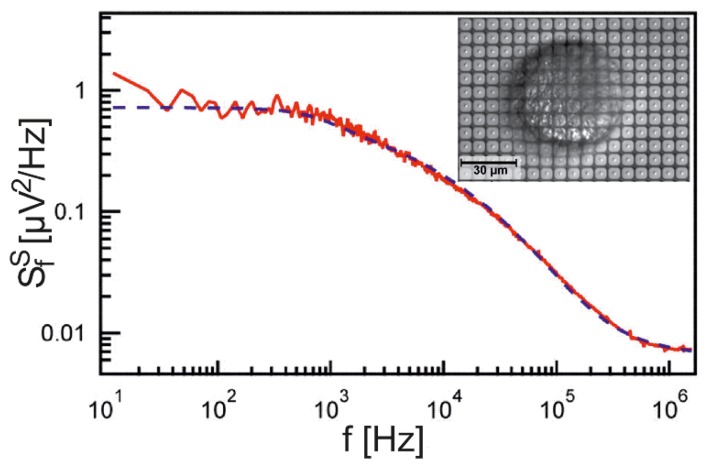
Power spectral density (PSD) of the fluctuating extracellular voltage *V_J_* in a cell–substrate junction (by permission [26]). The PSD of a dissociated neuron from *Lymnaea stagnalis* is probed with an array of EOMOS transistors (insert). The logarithm of the net PSD per frequency interval for the sensor transistor in the center of the adhesion area (after subtracting the PSD without cell) is plotted versus the logarithm of the frequency (red trace). The data are fitted by the PSD of the thermal voltage fluctuations in a planar capacitive core-coat conductor with a sheet resistance *r_J_* = 109 *M*Ω and a capacitance (whole cell) of *c_J_* = 0.74 *µF/cm^2^* (blue dashed trace).

The PSD for a wide range of frequencies has been probed for neurons from *Lymnaea stagnalis* using an array of EOMOS transistors, as shown in Figure 3 [26]. The data are fitted with a sheet resistance $r_{J}=109M\Omega$ and a junction capacitance (whole cell) of $c_{J}=0.74\;{\mu F/cm}^{2}$. The unusually large sheet resistance, which indicates a particularly close adhesion, was due to a special preparation of the dissociated snail neurons. The perfect fit of the data over five orders of magnitude of frequencies confirms that the cell-substrate junction is indeed a capacitive core-coat conductor.

## 5. One-Compartment Model

The typical issue of a cell-semiconductor assembly is the electrical coupling of the ionic and the electronic system across the planar junction. The profiles of the electrical potential and of the ion concentrations are not so important. For that reason, the planar core-coat conductor can be drastically reduced: drift and diffusion of ions along the differential area elements of the junction are replaced by global drift and diffusion from a single compartment to the bath electrolyte. On the one hand, this simple approach provides a surprisingly good approximation for the dynamics of the planar core-coat conductor, and, on the other hand, it is adequate if a cell-substrate junction is not sufficiently well defined with respect to the profiles of resistance, capacitance, and membrane conductance and to the position of a transistor. The “one-compartment model” is derived from the planar core-coat conductor for constant ion concentrations as well as for changing concentrations.

*One*-*compartment model for constant ion concentrations.* We start with the 2D cable equation (Equation (9)) for a homogeneous circular junction (Equation (11)). We replace the divergence of the electrical current by a global current from a junction compartment to the bath according to Ohm’s law (formally by a substitution $-\nabla^{2}\Rightarrow \eta_{J}/A_{J}$) and obtain Equation (21a).

(21a) $$g_{J}V_{J}+c_{J}\frac{dV_{J}}{dt}=c_{M}\frac{dV_{M}}{dt}+\sum g_{JM}^{i}\left(V_{M}-V_{J}-V_{M0}^{i}\right)$$

(21b) $$g_{J}=\eta_{J}\frac{d_{J}}{A_{J}\rho_{J}}$$

The Ohmic conductance per unit area $g_{J}$ as defined by Equation (21b) combines three crucial parameters of a cell-substrate junction, the distance $d_{J}$, the area $A_{J}$, and the resistivity $\rho_{J}$. The scaling factor $\eta_{J}$ is obtained by comparing the one-compartment model with a circular core-coat conductor as follows.

When a voltage step $\Delta V_{M}^{\;0}$ is applied to a cell without membrane conductance, the capacitive current induces a junction voltage, which decays according to Equation (22a). Starting from $V_{M}=V_{M0}^{i}$ for a single ion conductance $g_{JM}^{i}$, the voltage step gives rise to an increasing junction voltage according to Equation (22b) for $\left|V_{J}\right|<<\left|V_{M}-V_{M0}^{i}\right|$ with a stationary voltage $\Delta V_{M}^{\;0}g_{JM}^{i}/g_{J}$.

(22a) $$V_{J}^{}=\Delta V_{M}^{0}\left(c_{M}/c_{J}\right)\exp\left(-t\;g_{J}/c_{J}\right)$$

(22b) $$V_{J}^{}=\Delta V_{M}^{0}\left(g_{JM}^{i}/g_{J}\right)\left[1-\exp\left(-t\;g_{J}/c_{J}\right)\right]$$

When the time constant $\tau_{J}=c_{J}/g_{J}$ is compared with the time constant $\tau_{J}^{\left(1\right)}$ of the circular core-coat conductor (Equation (12)), we obtain $\eta_{J}=5.78\;\pi$. When the stationary voltage is compared with the stationary parabolic voltage profile (Equation (13)), the scaling factor is $\eta_{J}=4\;\pi$ or $\eta_{J}=8\;\pi$, respectively, if the maximum voltage or the average voltage is considered.

The one-compartment model for constant ion concentrations corresponds to a simple equivalent electrical circuit with a single node assigned to the cell-substrate junction and an Ohmic seal conductance [27]. The formal equivalent circuit, however, does not provide a relation of the seal conductance with the structural parameters of a cell-substrate junction as it is obtained when the model is derived from the planar core-coat conductor [28].

*One*-*compartment model with changing ion concentrations.* A one-compartment model can be also defined for changing ion concentrations [13]. We start with the drift-diffusion equation (Equation (6)) for a homogeneous junction. The divergence of the ion flux is replaced by the global flux from a junction compartment to the bath with Ohm’s law for the drift and with Fick’s first law for the diffusion. We obtain Equation (23a), where the ion conductivity $1/\rho_{J}^{i}$ and reversal voltage $V_{JM0}^{i}$ for the adherent membrane depend on the ion concentration according to Equations (23b) [29].

(23a) $$d_{J}z_{i}e_{0}\frac{dc_{J}^{i}}{dt}+\frac{\eta_{J}d_{J}}{A_{J}\rho_{J}^{i}}\left[\left(\varphi_{J}-\varphi_{E}^{\infty }\right)\;+\frac{k_{B}T}{z_{i}^{}e_{0}^{}}\frac{1}{c_{J}^{i}}\left(c_{J}^{i}-c_{E}^{i}\right)\right]=g_{JM}^{i}\left(V_{M}-V_{J}-V_{JM0}^{i}\right)$$

(23b) $$\frac{1}{\rho_{J}^{i}}=z_{i}^{2}e_{0}^{2}u_{J}^{i}c_{J}^{i},\;V_{JM0}^{i}=\frac{k_{B}T}{z_{i}e_{0}}\ln\frac{c_{J}^{i}}{c_{cell}^{i}}$$

The voltage $V_{J}=\varphi_{J}-\varphi_{E}^{\infty }$ is related with the total electrical charge according to the differential capacitor relation of Equation (24a) (at a constant bias voltage of the semiconductor). For a constant membrane voltage, the integral capacitor relation of Equation (24b) can be used considering an initial condition $c_{J}^{i}\left(t=0\right)=c_{E}^{i}$ and the electroneutrality condition $\sum z_{i}e_{0}c_{E}^{i}\;=0$ for the bath electrolyte.

(24a) $$\sum d_{J}z_{i}e_{0}\frac{dc_{J}^{i}}{dt}\;=c_{J}\frac{dV_{J}}{dt}-c_{M}\frac{dV_{M}}{dt}$$

(24b) $$\sum d_{J}z_{i}e_{0}c_{J}^{i}\;=c_{J}V_{J}$$

## 6. Transistor Recording of Ion Conductances

Voltage-activated membrane conductances are crucial for the transistor sensorics of electrogenic cells. As examples, we consider the inactivating sodium conductance and the non-inactivating potassium conductance of the classical Hodgkin-Huxley (HH) model [30]. They are described by maximum conductances $g_{M}^{\infty \;Na}$ and $g_{M}^{\infty \;K}$ and by the open probabilities of independent gates p=m,n for activation and p=h for inactivation according to Equation (25a). The open probabilities obey rate equations (Equation (25b)) with the rate constants $\alpha_{p}$ and $\beta_{p}$, which depend on the transmembrane voltage.

(25a) $$g_{M}^{Na}=g_{M}^{\infty \;Na}m^{3}h,\;g_{M}^{K}=g_{M}^{\infty \;K}n^{4}$$

(25b) $$dp/dt=\alpha_{p}\left(1-p\right)-\beta_{p}p$$

*Quasi*-*stationary response.* The time constant $\tau_{p}$ for the dynamics of the gates at common transmembrane voltages is in a range of 0.1–1 *ms* for the activation of the Na conductance and in a range of 1–10 *ms* for the inactivation of the Na conductance and the activation of the K conductance. Thus, the dynamics is far slower than the electrical relaxation of the cell-substrate junction but faster than the ion diffusion in the junction. The relation $\tau_{J}<<\tau_{p}<\tau_{J,diff}$ has two consequences: (i) the junction voltage follows the membrane current in a quasi–stationary state; and (ii) the change of the extracellular ion concentrations due to the inactivating Na conductance is small, whereas it may be large for the non-inactivating K conductance if it is held open by a constant intracellular voltage.

To elucidate the transistor response of ion channels, the voltage in an adherent cell is controlled by a whole-cell patch-pipette. With this approach a transistor signal $\delta V_{Gpp}^{EOS}$ can be compared with the total current through the cell membrane $I_{M}$ such that a current-induced and an interface-induced responses can be distinguished. As a guide, various numerical simulations of the capacitive core-coat conductor are performed with the HH conductances (rate constants at 18.5 °C) using the one-compartment model with changing ion concentrations according to Equations (23) and (24a) with an area $A_{J}=200\;\mu m^{2}$, a distance $d_{J}=70\;nm$, and tabulated diffusion coefficients. A scaling factor *η_J_* = 5.78π takes into account the partial spatial averaging by a transistor [31].

*Voltage*-*clamp with constant ion concentrations.* Almost constant ion concentrations in the junction are expected for the Na conductance due to the fast inactivation and the high extracellular Na concentration. This assumption is confirmed by a simulation with $g_{JM}^{\infty \;Na}=20\;mS/cm^{2}$ (extracellular electrolyte 50 *mM*·NaCl, 2 *mM*·KCl, intracellular electrolyte 12 *mM* Na ions).

Considering Equation (21), the quasi-stationary junction voltage is described by Equation (26a) for $\left|V_{J}\right|<<\left|V_{M}-V_{M0}^{Na}\right|$ at a constant intracellular voltage. The current through the whole membrane with an area $A_{M}$ and an average conductance ${\bar{g}}_{M}^{Na}$ is expressed by Equation (26b).

(26a) $$V_{J}\left(t\right)=\frac{1}{g_{J}}g_{JM}^{Na}\left(t\right)\cdot \left[V_{M}-V_{M0}^{Na}\right]$$

(26b) $$I_{M}\left(t\right)=A_{M}{\bar{g}}_{M}^{Na}\left(t\right)\cdot \left[V_{M}-V_{M0}^{Na}\right]$$

If the dynamics of the conductance is the same in the whole membrane, the voltage $V_{J}$ and the current $I_{M}$ exhibit the same waveform and are proportional according to Equation (27).

(27) $$V_{J}=\frac{1}{g_{J}A_{M}}\frac{g_{JM}^{\infty \;Na}}{{\bar{g}}_{M}^{\infty \;Na}}I_{M}$$

**Figure 4:**
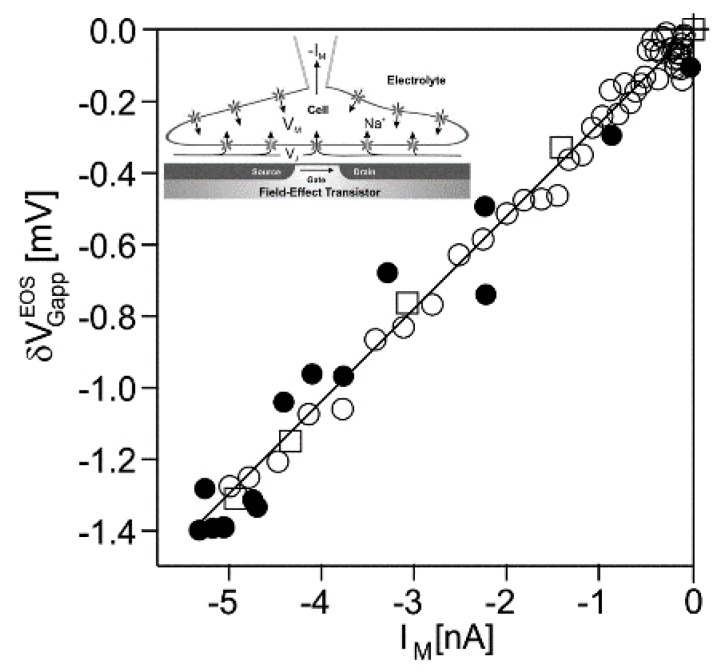
Transistor recording of the sodium channel Na_v_1.4 in a HEK293 cell at constant intracellular voltage (by permission [32]). The apparent change of the gate voltage $\delta V_{Gapp}^{EOS}$ is plotted versus the membrane current $I_{M}$ upon a depolarization from *–*90 *mV* to *–*20 *mV*. (Scheme: cell with whole-cell patch-pipette). The white squares refer to the activation phase and the white circles to the inactivation phase. In addition, the minima of the voltage and of the current transients are plotted for 15 different depolarizations (black dots). The data are fitted by the relation $\delta V_{Gapp}^{EOS}=260\;k\Omega \cdot I_{M}$.

*Na_v_1.4 channel.* Transistor recordings under voltage–clamp have been reported for the sodium channel Na_v_1.4 in HEK293 cells at an extracellular Na concentration of 50 *mM* [32]. The Na conductance was activated by depolarizing steps of the intracellular voltage. The transistor response $\delta V_{Gapp}^{EOS}$ and the membrane current $I_{M}$ reflected both the typical waveform of a transient sodium current with an activation phase and an inactivation phase. The apparent change of the gate voltage is plotted in Figure 4 versus the total membrane current. There is a perfect proportionality, which is fitted with a relation $\delta V_{Gapp}^{EOS}=260\;k\Omega \cdot I_{M}$. The result indicates that the transistor signal is indeed due to a pure current-induced response with $\delta V_{Gapp}^{EOS}=V_{J}$. By ac stimulation and recording the response of transistor and current, a parameter $1/g_{J}A_{M}=308\;k\Omega$ has been measured. Considering Equation (27), a depleted conductance in the adherent membrane with $g_{JM}^{\infty \;Na}=0.85\;{\bar{g}}_{M}^{\infty \;Na}$ was estimated.

**Figure 5:**
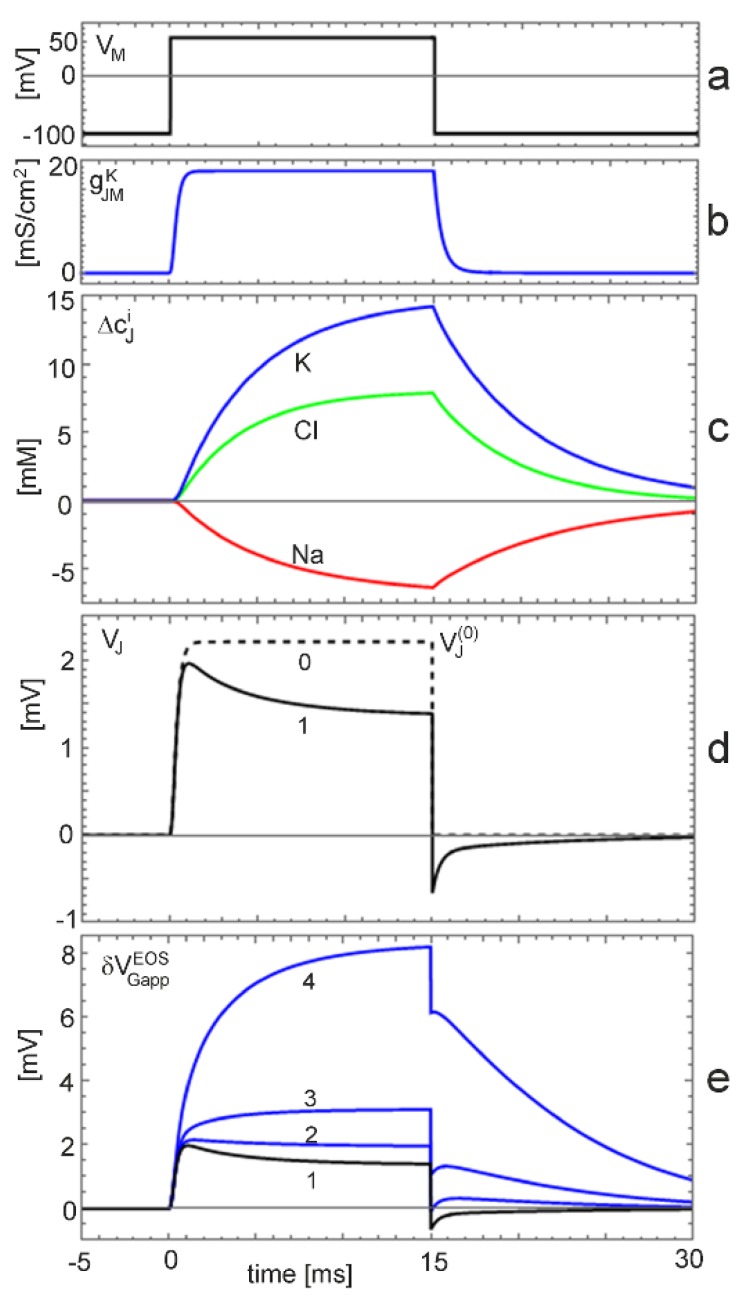
Computed dynamics of a cell-substrate junction for a non-inactivating K conductance under voltage-clamp at a low extracellular K concentration. One-compartment model of the capacitive core-coat conductor with the K conductance of the Hodgkin-Huxley model. For the parameters, see text. (**a**) Intracellular voltage $V_{M}$; (**b**) membrane conductance $g_{JM}^{K}$ ; (**c**) changes $\Delta c_{J}^{i}=c_{J}^{i}-c_{E}^{i}$ of extracellular concentrations; (The relative change $\Delta \;c_{J}^{i}/c_{E}^{i}$ is high for K with $c_{E}^{K}=5\;mM$ but low for Na and Cl with $c_{E}^{Na}=135\;mM$ and $c_{E}^{Cl}=140\;mM$). (**d**) current–induced voltage for constant ion concentrations (trace 0) and for changing ion concentrations (trace 1); and (**e**) transistor response for a gate oxide without ion sensitivity (trace 1), and with ion sensitivities of 1 *mV* (trace 2), 3 *mV* (trace 3), and 12 *mV* (trace 4) (all traces without capacitive transients).

*Voltage*-*clamp with changing ion concentration.* Changing ion concentrations are expected under voltage-clamp for the non-inactivating K conductance at the low physiological K concentration. The situation is illustrated by a simulation with $g_{JM}^{\infty \;K}=20\;mS/cm^{2}$ (extracellular electrolyte 5 *mM*·KCl, 135 *mM*·NaCl, intracellular electrolyte 40 *mM* K ions). The cell is depolarized by 140 *mV* for 15 *ms* starting from the reversal voltage of potassium. The results are plotted in Figure 5. We have to distinguish three levels: (i) the current-induced response for constant ion concentrations; (ii) the effect of the ion concentrations on the current-induced response; and (iii) the additional effect of the ion concentrations on the interface-induced response.

The junction voltage for constant ion concentrations is plotted in Figure 5d as trace 0. Upon depolarization, a stationary level of $V_{J}^{\left(0\right)}=2.2\;mV$ is reached within a millisecond. The dynamics is determined by the activation of the potassium conductance (Figure 5b). The stationary voltage disappears suddenly upon repolarization to the reversal voltage.

The junction voltage with changing ion concentrations is shown in Figure 5d as trace 1. Within a millisecond, it reaches almost the level of $V_{J}^{\left(0\right)}$. It relaxes within several milliseconds to a stationary voltage of $V_{J}^{}=1.4\;mV$. This decay corresponds to the dynamics of the ion concentrations (Figure 5c). Upon repolarization, the voltage drops suddenly to a negative level and relaxes in a fast phase due to the dynamics of the ion conductance and in a slow phase due to the dynamics of the concentrations.

The K concentration in the junction increases from 5 *mM* to 15 *mM* (Figure 5c). The logarithmic change $\log\;c_{J}^{K}/c_{E}^{K}\approx 0.5$ may give rise to an interface-induced response (Equation (10)). The total transistor signal $\delta V_{Gapp}^{EOS}$ is plotted in Figure 5e for an insensitive gate (trace 1) with $\delta V_{Gapp}^{EOS}=V_{J}$ and for three ion sensitivities $S_{GJ}^{K}$. For a low sensitivity of 1 *mV* (trace 2), the interface-induced response compensates partially the ion-dependent dynamics of the current-induced response upon depolarization and repolarization. For a sensitivity of 3 *mV* (trace 3), the transistor signal exhibits two positive components upon depolarization, a fast response, which is determined by the dynamics of the conductance, and a slow response, which is determined by the dynamics of the K concentration. Upon repolarization, the transistor signal drops suddenly due to the disappearance of the membrane current. Then, the interface-induced response relaxes with the ion concentrations. For an ion sensitivity of 12 *mV* (trace 4), the current-induced and the interface-induced response are fused to a smooth, large signal up to $\delta V_{Gapp}^{EOS}=8.5\;mV$. The interface-induced component is six times larger than the current–induced response. Upon repolarization, the signal drops suddenly due to the disappearance of the membrane current. Then, the interface-induced response relaxes with $\log\;c_{J}^{K}$.

Transistor recordings of non–inactivating K conductances under voltage clamp have been reported for hippocampal neurons [33] and for HEK293 cells with the recombinant channels Maxi-K [12,34], Kv1.3 [13,35], and EAG [16].

**Figure 6:**
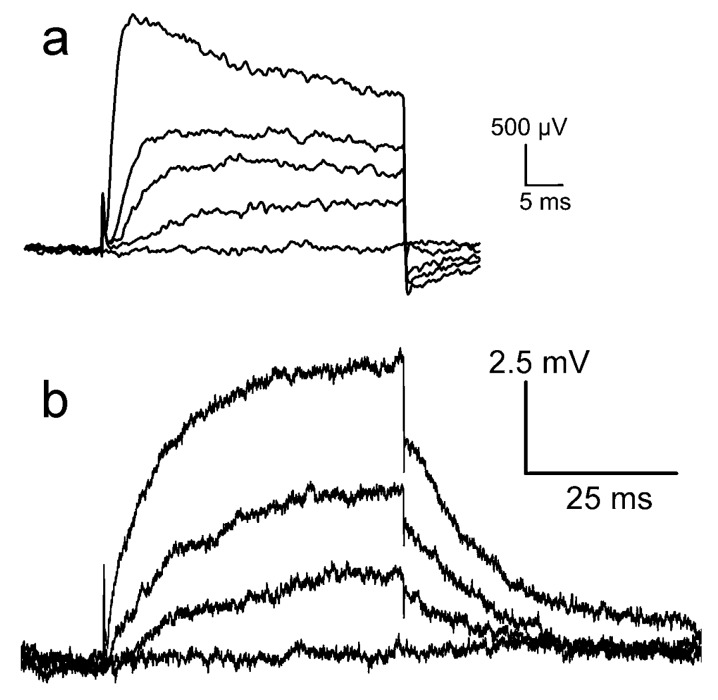
Transistor recordings of the potassium channel Kv1.3 in HEK293 cell at a constant intracellular voltage with an extracellular K concentration of 5 *mM*. The apparent change of the gate voltage $\delta V_{Gapp}^{EOS}$ is plotted versus time. (**a**) Gate oxide cleaned with an acidic detergent: depolarizations to −25, −15, −5, and +40 *mV* starting near the reversal voltage of potassium, repolarization after 40 *ms* [35]; (**b**) Gate oxide cleaned with an alkaline detergent: depolarizations to −20, 0, and +50 *mV*, repolarization after 50 ms (by permission of Springer [13]). The conditions of the experiments differ with respect to channel expression, cell size, and transistor quality. A comparison can refer only to the dynamics and the relative amplitudes of the fast and slow components.

*Kv1.3 channel.* The Kv1.3 channel has been studied on chips that were cleaned with an alkaline detergent [13] and with an acidic detergent [35]. Recordings of the “acidic experiment” are plotted in Figure 6a. Upon depolarization, an effective change of the gate voltage up to about 2.5 *mV* appears within 2 *ms*. It decays partially during depolarization. Upon repolarization, the signal drops suddenly and a transient negative signal appears. Recordings of the “alkaline experiment” are displayed in Figure 6b, wherein a smooth increase of the transistor signal up to 7.5 *mV* is observed. Upon repolarization, the signal drops suddenly by 2 *mV*, and the remaining amplitude relaxes slowly. (The conditions of the two experiments differ with respect to channel expression, cell size, and transistor quality; the results can be compared only with respect to the dynamics and the relative amplitudes of the fast and slow components).

For the same ion channel, the waveforms of the transistor signals are rather different. When we compare the highest traces of Figure 6a,b with the simulation of Figure 5e, it is apparent that the waveform of the “acidic experiment” resembles trace 1 for a pure current*–*induced response with $\delta V_{Gapp}^{EOS}=V_{J}$, whereas the waveform of the “alkaline experiment” resembles trace 4 with a superposed interface-induced response for an ion sensitivity 12 *mV* of the gate. The different ion sensitivities must be related with the cleaning procedures. In fact, after cleaning with the alkaline detergent, the chip surface was highly hydrophilic, whereas it was extremely hydrophobic after cleaning with the acidic detergent due to the deposition (selfassembly) of a cationic amphiphile, which blocks the negative charges of silica.

It appears that rather complex transistor signals can be described in terms of a capacitive core-coat conductor with changing ion concentrations. However, two problems must be considered.

(i) The core-coat conductor theory is not able explain the transistor recordings in terms of the cellular currents with a changing extracellular voltage and changing ion concentrations as long as the ion sensitivity of the gate in the cell-substrate junction is unknown. A calibration of a cell-free transistor is not adequate. For example, the sensitivity $S_{GJ}^{K}=12\;mV$ that matches the “alkaline experiment” is distinctly lower than the sensitivity $S_{GE}^{K}\approx 45\;mV$ that was obtained by titration [13]. The discrepancy must be assigned to a modification of the chip surface by cell adhesion such that negative oxide charges are partially compensated by adsorbed poly-lysine and extracellular matrix protein. (Such a compensation of negative surface charges by a cationic polymer had been observed for a monomolecular lipid film using a fluorescent probe [36]).

(ii) The pattern of the transistor signals (Figure 6) is matched quite nicely by the computations (Figure 5e). However, the interface-induced response is slower than the computed dynamics of the ion concentrations. A low ion mobility in the electrolyte film of the cell-substrate junction can be excluded because the resistivity in the junction is the same as in the bulk electrolyte [24]. The effect may be due to a slow kinetics of ion binding [13,37].

*EAG channel*. Current-induced and interface-induced signals have been also distinguished in transistor recordings of the EAG potassium channel under voltage-clamp [16]. The current-induced response was low around 0.2 *mV* due to a low membrane current. Also the interface-induced response was low due to a low ion sensitivity of 0.7 *mV* of the gate (maybe caused by a cleaning procedure with an incubation in 20% H_2_SO_4_). The data were explained in terms of the one-compartment model with constant ion concentrations for the current-induced response and by a Poisson-Nernst-Planck approach to compute the changed ion concentrations, which gave rise to the interface-induced response (see Appendix C).

**Figure 7:**
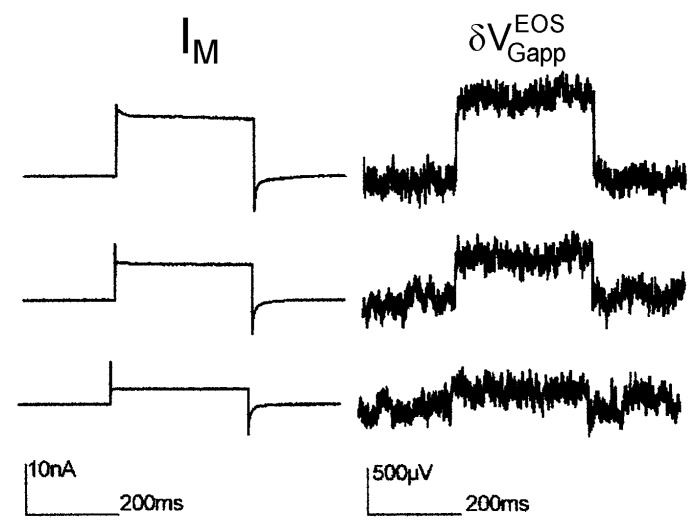
Transistor recording of Maxi-K potassium channel in HEK293 cell at a constant intracellular voltage with a high extracellular K concentration of 100 *mM* [34]. (**Left**) Total membrane current; (**Right**) Transistor recording. Depolarizations from −100 *mV* to 20, 45, and 58 *mV*.

*Maxi-K channel with high extracellular K concentration*. In a first report on transistor recording of a recombinant ion channel (Maxi-K) under voltage clamp [34], a high extracellular potassium concentration of 100 *mM* has been applied. The intention was to suppress the interface-induced response, which had been observed when the transistor was cleaned with the alkaline detergent [12]. The results are displayed in Figure 7. Upon depolarization, the noisy signals with low time resolution indicate stationary transistor signaly up to 1 *mV*, which are proportional to the total membrane current with $\delta V_{Gapp}^{EOS}=73\;k\Omega \cdot I_{M}$.

A simulation (extracellular electrolyte 100 *mM*·KCl, 35 *mM*·NaCl, intracellular electrolyte 140 *mM* K ions) with an ion sensitivity of 12 *mV* for the gate oxide yields a biphasic waveform of the transistor signal that resembles trace 3 in Figure 5e with a fast current-induced response $V_{J}^{\left(0\right)}=0.85\;mV$ and a stationary signal $\delta V_{Gapp}^{EOS}=1.05\;mV$ that is reached within several milliseconds. Thus the high extracellular K concentration lowers drastically the interface-induced component (*cf.* Figure 5e), but does not abolish it completely. On the basis of the simulation, a relation $V_{J}^{\left(0\right)}=60\;k\Omega \cdot I_{M}$ is estimated for the current-induced component of the Maxi-K recording. With a weighting factor $1/g_{J}A_{M}=22\;k\Omega$ (measured by ac stimulation), an enhancement of the Maxi-K conductance in the adherent membrane of $g_{JM}^{\infty \;K}=2.7\;{\bar{g}}_{M}^{\infty \;K}$ is obtained using Equation (27) (in comparison to a value of 3.4 estimated in [34]).

**Figure 8:**
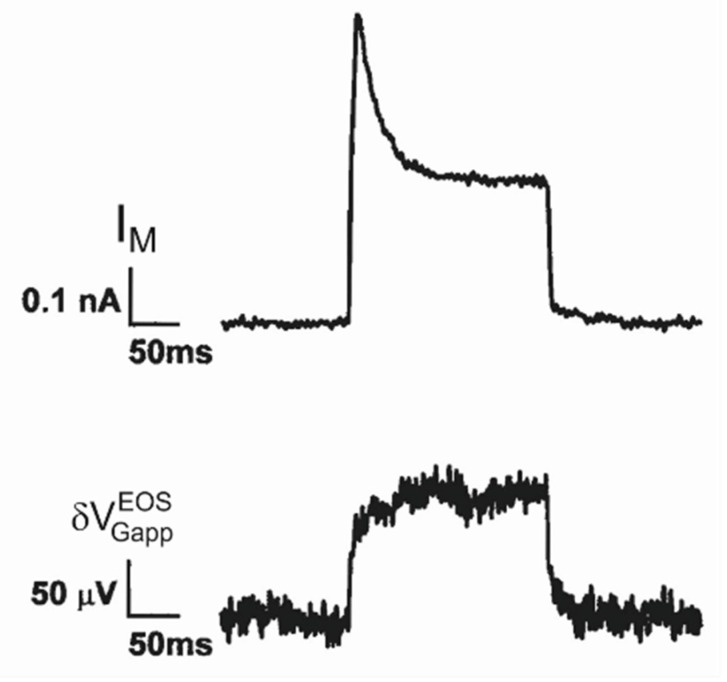
Transistor recording of potassium conductances in cultured hippocampal rat neuron at a constant intracellular voltage [33]. (**Top**) Total membrane current; (**Bottom**) Transistor recording. After a prepolarization for 200 *ms* to −110 *mV*, the cell was depolarized to 20 *mV* and repolarized to −50 *mV*. The current exhibits an inactivating A-type potassium conductance and a non-inactivating K-type potassium conductance. The transistor signal is assigned to the K-type conductance with a fast current-induced response and a slow component due to the changing potassium concentration. There is no transistor response to the A-type conductance.

*K conductance in hippocampal neurons.* A first transistor recording under voltage-clamp with voltage-activated potassium conductances has been reported for cultured hippocampal neurons [33]. The membrane current and the transistor signal are plotted in Figure 8. (The chips were cleaned with a mild dish detergent). Upon depolarization the membrane current exhibits an inactivating A-type potassium conductance and a non-inactivating K-type potassium conductance. The transistor response is quite different. The apparent change of the gate voltage increases rapidly by about 80 *µV* and further more slowly to a stationary level of 120 *µV*. Upon deactivation, the voltage drops suddenly by about 80 *µV* with a subsequent slow relaxation. The fast and slow signal were not distinguished in [33].

It appears that the transistor does not record an A*-*type conductance in the junction at all, whereas it responds to the non*–*inactivating K*-*type conductance with a biphasic waveform that resembles again trace 3 in Figure 5e. Attributing the fast signal to a current-induced response $V_{J}^{\left(0\right)}=80\;\mu V$ and considering the stationary membrane current $I_{M}=0.25\;nA$, we obtain a relation $V_{J}^{\left(0\right)}=320\;k\Omega \;\cdot I_{M}$. With a weighting factor $1/g_{J}A_{M}=90\;k\Omega$ (obtained from a capacitive transient), an enhancement of the K-type conductance in the adherent membrane $g_{JM}^{\infty \;K,K}=3.5\;{\bar{g}}_{M}^{\infty \;K,K}$ is obtained using Equation (27) (in comparison to a value of 5.7 estimated in [33]), whereas the A-type conductance in the adherent membrane is low with $g_{JM}^{\infty \;K,A}<<\;{\bar{g}}_{M}^{\infty \;K,A}$.

## 7. Transistor Recording of Neuronal Action Potentials

An action potential (AP) in neurons arises from the dynamics of the membrane voltage and of voltage-activated conductances. In the Hodgkin-Huxley model, the AP is an almost monophasic positive transient $V_{M}\left(t\right)$ of the membrane voltage with a fast rise due to a Na inward current, a slower decay due to a K outward current with an inactivating Na current, and a minor negative tail [30]. Two issues must be considered when transistors are applied for extracellular recording of APs in an intact cell: (i) the dynamics of the voltage in the closed cell must be taken into account; and (ii) a local compensation of the ionic and capacitive currents in the adherent membrane must be avoided.

*Current compensation.* Let us consider a free neuron with a homogeneous membrane in a homogeneous electrolyte. The global current balance for the whole cell (membrane area $A_{M}$) with homogeneous ion conductances $g_{M}^{i}$ is described by Equation (28).

(28) $$A_{M}\left[\sum g_{M}^{i}\left(V_{M}-V_{0}^{i}\right)+c_{M}\frac{dV_{M}}{dt}\right]=0$$

The global current balance implies a local compensation of the ionic and capacitive currents in every area element of the membrane. There is no current flow to the surrounding electrolyte and no extracellular voltage that could be recorded. To obtain an extracellular voltage, the membrane must be inhomogeneous with an enhanced current in one region and a depleted current in another region. For a homogeneous capacitance, that inhomogeneity is created by an inhomogeneous distribution of ion conductances, which is given by the intrinsic structure of a cell or induced by the interaction with the substrate.

*Current balance for an adherent cell.* We consider a two*–*domain model of the cell membrane with ion conductances $g_{FM}^{i}$ in the free membrane (area $A_{F}$) and ion conductances $g_{JM}^{i}$ in the adherent membrane (area $A_{J}$). Considering that the conductances are activated for a short time during a neuronal AP, the effect of changing extracellular ion concentrations is disregarded. We use the one-compartment model for constant ion concentrations according to Equation (21) with a quasi-stationary dynamics of the junction voltage. For small voltages $\left|V_{J}\right|<<\left|V_{M}-V_{M0}^{i}\right|$, we obtain Equation (29a). The current balance of the closed cell is expressed by Equation (29b).

(29a) $$\sum g_{JM}^{i}\left(V_{M}-V_{M0}^{i}\right)+c_{M}\frac{dV_{M}}{dt}=g_{J}V_{J}$$

(29b) $$\sum \left(A_{F}g_{FM}^{i}+A_{J}g_{JM}^{i}\right)\left(V_{M}-V_{M0}^{i}\right)+\left(A_{F}+A_{J}\right)c_{M}\frac{dV_{M}}{dt}=0$$

Two simple models are considered for inhomogeneous ion conductances with $g_{JM}^{i}\neq g_{FM}^{i}$ in the adherent and free membrane.

*Leak conductance in soma*-*substrate junction*. In the first model the cell-substrate junction is formed by part of a neuronal soma with low voltage-activated conductances. The interaction with the substrate is assumed to induce a leak conductance $g_{JM}^{leak}$ with $V_{M0}^{leak}=0$. The junction voltage is determined by the leak current and the capacitive current through the adherent membrane according to Equation (28a). We obtain Equation (30) when we give up the condition $\left|V_{J}\right|<<\left|V_{M}\right|$.

(30) $$V_{J}=\frac{1}{g_{J}+g_{JM}^{leak}}\left(g_{JM}^{leak}V_{M}+c_{M}\frac{dV_{M}}{dt}\right)$$

For a low leak conductance, the junction voltage is dominated by the capacitive current and proportional to the first derivative of the intracellular voltage. If an almost monophasic AP is created by voltage-activated conductances in the free membrane (*e.g.*, in the axon hillock), a biphasic positive-negative transient $V_{J}\left(t\right)$ is induced (A-type response). If the leak conductance in the junction is large, the voltage transient $V_{J}\left(t\right)$ is proportional to the intracellular voltage, and the response to an AP is monophasic positive (B-type response).

In fact, two kinds of transistor signals had been observed in the original transistor recordings of action potentials for adhering leech neurons [38] with a small response $\delta V_{Gapp}^{EOS}$ resembling $dV_{M}/dt$ and a large response $\delta V_{Gapp}^{EOS}$ resembling $V_{M}\left(t\right)$. The interpretation of these recordings in terms of an A-type and B-type response of the leak model was given in a subsequent paper [39]. It was illustrated by a test experiment.

**Figure 9:**
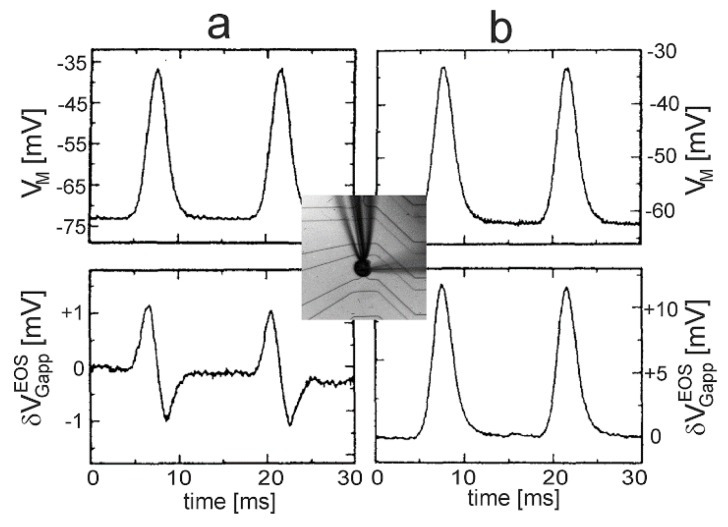
Transistor recording of leech neurons upon stimulation by a Gaussian transient of the intracellular voltage [39]. (**a**) Soma-substrate junction with low leak conductance; (**b**) Soma-substrate junction with an enhanced leak conductance and a reduced junction conductance. The upper graphs display the intracellular voltage $V_{M}\left(t\right)$, and the lower graphs the apparent change of the gate voltage $\delta V_{Gapp}^{EOS}\left(t\right)$. The micrograph shows a leech neuron on an EOSFET with a whole-cell patch-pipette used to apply the Gaussian voltage transients and with an impaled micropipette used to record the intracellular voltages.

Defined Gaussian transients $V_{M}\left(t\right)$ with σ=1.25 ms were applied to leech neurons using a whole-cell patch-pipette. A-type and B-type responses of the transistor signal $\delta V_{Gapp}^{EOS}$ were observed for different cells, as shown in Figure 9 [40]. The two waveforms can be parametrized according to Equation (30) for the A-type response with $g_{JM}^{leak}=0.1\;mS/cm^{2}$, $g_{J}^{}=25\;mS/cm^{2}$ and for the B-type response with an enhanced leak conductance $g_{JM}^{leak}=5\;mS/cm^{2}$ (responsible for a changed waveform) and a reduced junction conductance $g_{J}^{}=10\;mS/cm^{2}$ (responsible for a larger amplitude).

Reversible transitions between A-type and B-type recordings of action potentials have been observed when neurons were pressed toward the substrate and when the force was released again [41,42]. These transitions were connected with enhanced/reduced leak conductance and a reduced/enhanced junction conductance, respectively. The mechanism is not clear.

*Depleted/enhanced voltage-activated conductances*. In the second model all voltage-activated conductances in a neuronal membrane are depleted or enhanced in the cell-substrate junction by the same factor $\mu_{J}$ with $g_{JM}^{i}=\mu_{J}\;g_{FM}^{i}$. When all ion currents are eliminated from Equations (28a) and (28b), considering Equation (21b), the striking Equation (31) is obtained as a relation between the extracellular and the intracellular voltage [43].

(31) $$V_{J}=\frac{\left(1-\mu_{J}\right)A_{J}A_{F}}{A_{F}+\mu_{J}A_{J}}\frac{r_{J}c_{M}}{\eta_{J}}\frac{dV_{M}}{dt}$$

For complete depletion ($\mu_{J}=0$), the junction voltage is determined by the capacitive current through the adhesion area $A_{J}$. For a homogeneous membrane ($\mu_{J}=1$), there is no response at all due to local current compensation. For strong enhancement ($\mu_{J}>>1$), the junction voltage is determined by the ionic current through $A_{J}$, which appears in Equation (31) as a negative capacitive current through $A_{F}$. The first limit corresponds to a soma–substrate junction without leak conductance as considered above (biphasic positive-negative response to an AP). The second limit corresponds to a junction where the local ion currents dominate with a biphasic negative-positive response to an AP.

**Figure 10:**
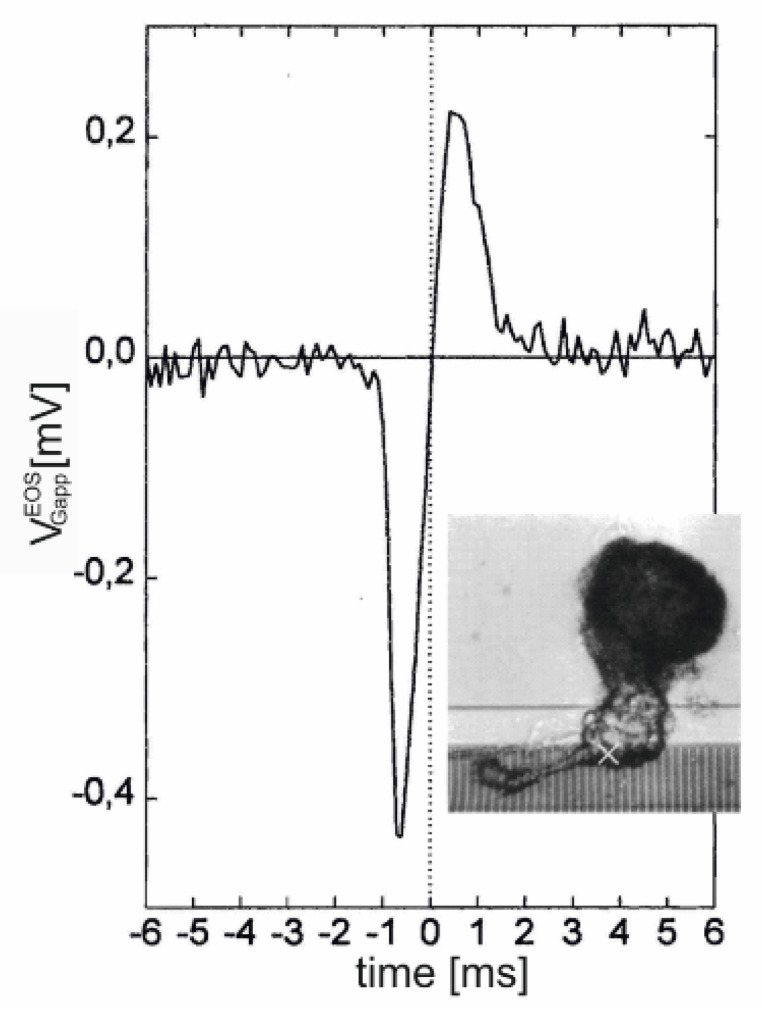
Transistor recording of an action potential beneath the axon stump of a leech neuron (by permission [44]). The apparent change of the gate voltage $\delta V_{Gapp}^{EOS}$ was observed with the transistor of a linear array as marked in the micrograph. The biphasic negative-positive signal reflects the extracellular voltage $V_{J}=\delta V_{Gapp}^{EOS}$ as induced by the local ion currents in the cell-substrate junction.

Dissociated leech neurons have been attached with their axon stump to an array of EOSFETs [44], and an AP was elicited with an impaled micropipette. A biphasic negative-positive transistor signal was observed, as shown in Figure 10. The current-induced response $\delta V_{Gapp}^{EOS}=V_{J}$ is induced by the large local ion currents in the axon stump. Due to the global current balance in the cell, the signal reflects the inverted capacitive current through the soma membrane. The asymmetry of the biphasic $V_{J}\left(t\right)$ with a deeper trough corresponds to the asymmetry of an almost monophasic $V_{M}\left(t\right)$.

Various waveforms of transistor recordings of APs were reported for cultured hippocampal rat neurons [3,45] as well as for dissociated invertebrate neurons from *Lymnaea* [46] and *Aplysia* [47]. They were assigned to a selective enhancement and depletion of different voltage-activated conductances in the adherent membrane.

## 8. Transistor Recording of Propagating Action Potential

When an action potential propagates along an axon, the different components of the membrane current (capacitive, sodium, potassium) are separated in space. As a consequence, local membrane currents exist, even for a structurally homogeneous axon. An extracellular voltage is induced, which can be probed by transistor recording.

*Propagating AP.* The membrane current per unit area of an axon is balanced by the changing current along the core according to Equation (32) (cylinder radius $a_{M}$, core resistivity $\rho_{cell}$).

(32) $$g_{M}^{Na}\left(V_{M}-V_{M0}^{Na}\right)+g_{M}^{K}\left(V_{M}-V_{M0}^{K}\right)+c_{M}\frac{\partial V_{M}}{\partial t}=\frac{a_{M}}{2\rho_{cell}}\frac{\partial^{2}V_{M}}{\partial x^{2}}$$

**Figure 11:**
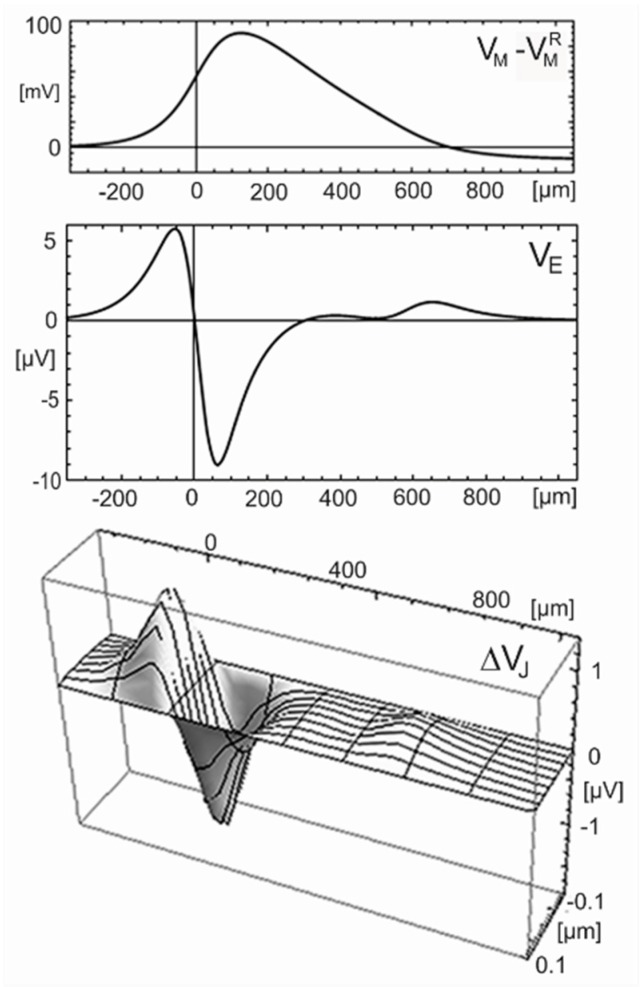
Computed voltage profiles along a cylindrical cable for a propagating action potential at an arbitrary time. (**Top**) Profile of the intracellular voltage with respect to the resting voltage $V_{M}\left(x\right)-V_{M}^{R}$ for the Hodgkin-Huxley model with a cable radius $a_{M}=0.5\;\mu m$. The action potential propagates in negative x-direction with a velocity of 0.86 *m/s*; (**Center**) Profile of the extracellular voltage $V_{E}\left(x,a_{M}\right)$ at the surface of the cable in a homogeneous electrolyte with aresistivity $\rho_{E}=100\;\Omega cm$; (**Bottom**) Profile of the extracellular voltage drop $\Delta V_{J}\left(x,y\right)$ in a cable-substrate junction with respect to the surrounding electrolyte for a lane–shaped junction (different scales along and across the junction). For the parameters, see text.

The coupling of the membrane voltage with the voltage*–*activated ion conductances gives rise to a wave $V_{M}\left(x\mp v_{AP}t\right)$ that propagates at a velocity $v_{AP}$ with an invariant waveform. The voltage profile at an arbitrary time is plotted in Figure 11 (top) as computed with the original Hodgkin-Huxley parameters $c_{M}=1\;\mu F/cm^{2}$, $\rho_{cell}=35.4\;\Omega cm$, $g_{M}^{\infty \;Na}=120\;mS/cm^{2}$, and $g_{M}^{\infty \;K}=36\;mS/cm^{2}$ (rate constants at 18.5 °C) for a radius $a_{M}=0.5\;\mu m$, typical for an unmyelinated mammalian axon. The velocity is $v_{AP}=0.86\;m/s$. The wave is almost monophasic with a steep rise and a smooth drop: The amplitude is 90 *mV* with a full width at half maximum of 260 *µm* in space and 300 *µs* in time. The voltage wave is connected with a current wave $i_{M}^{tot}\left(x\mp v_{AP}t\right)$, which is proportional to the curvature of the intracellular voltage in space as well as in time (because the voltage obeys a wave equation) according to Equation (33).

(33) $$i_{M}^{tot}\left(x,t\right)=\frac{a_{M}}{2\rho_{cell}}\frac{\partial^{2}V_{M}\left(x,t\right)}{\partial x^{2}}=\frac{a_{M}}{2\rho_{cell}}\frac{1}{v_{AP}^{2}}\frac{\partial^{2}V_{M}\left(x,t\right)}{\partial t^{2}}$$

This current wave induces a drop of the electrical potential in the surrounding. For a cable-substrate junction, the voltage with respect to the bulk electrolyte is partitioned into a voltage drop $\Delta V_{J}$ in the junction and a voltage $V_{E}$ in the surrounding electrolyte with $V_{J}=\Delta V_{J}+V_{E}$. We consider separately a cable in a homogeneous electrolyte and a cable-substrate junction.

*Homogeneous electrolyte.* The quasi–stationary response of the electrical potential in a volume conductor (resistivity $\rho_{E}$) to a current source density $i_{E}^{\;csd}$ is described by a 3D Poisson equation $-\nabla^{2}\varphi_{E}=\rho_{E}\;i_{E}^{\;csd}$ [19]. For a propagating AP, the contributions of all currents along the cylinder are superposed. The extracellular voltage $V_{E}\left(x,a,t\right)$ at a radial distance $a\geq a_{M}$ is given by Equation (34) when the membrane current per unit length $2\pi \;a_{M}\;i_{M}^{tot}\left(x,t\right)$ is placed along the cylinder axis [48]. (A logarithmic divergence of the integral is avoided due to the finite width of the current wave). A reaction of the extracellular voltage is neglected with $\left|V_{E}\right|<1\;mV$.

(34) $$V_{E}\left(x,a,t\right)=\frac{\rho_{E}}{4\pi }\int_{-\infty }^{+\infty }\frac{2\pi a_{M}\;i_{M}^{tot}\left(x?t\right)}{\sqrt{a_{}^{2}+{\left(x-x^{\prime }\right)}^{2}}}dx^{\prime }$$

The current $i_{M}^{tot}\left(x,t\right)$ is obtained from the Hodgkin-Huxley model for $a_{M}=0.5\;\mu m$. Equation (34) is evaluated with a resistivity $\rho_{E}=100\;\Omega cm$ typical for a culture medium. The profile of the extracellular voltage at the surface of the axon $V_{E}\left(x,a_{M},t\right)$ is plotted in Figure 11 (center). The wave is almost biphasic positive-negative and distinctly narrower than the monophasic wave of the intracellular voltage. Peak and trough are separated by 120 *µ*m in space and 140 *µs* in time. The amplitudes are 6 *µV* and −9 *µV* [49].

*Cable-substrate junction.* When a cylinder is attached to a planar substrate, a lane-shaped junction is formed with a distance $d_{J}$ that increases from a minimum $d_{J}^{0}$ at the midline towards the cylinder radius $a_{M}$. We replace that profile by a planar junction with a distance $d_{J}^{0}$ and match its resistance by choosing a half-width $y_{J}=\sqrt{2a_{M}d_{J}^{0}}$, where the actual distance would be $d_{J}=2d_{J}^{0}$. This planar junction is driven by the current $i_{M}^{tot}\left(x,t\right)$. The quasi-stationary response of the electrical potential is determined by a 2D Poisson equation $-\nabla^{2}\varphi_{J}=r_{J}\;i_{JM}^{tot}$ (Equation (14)). The curvature along the lane is neglected because the membrane current changes smoothly along the axon. The voltage drop $\Delta V_{J}\left(x,y,t\right)$ with a boundary condition $\Delta V_{J}\left(x,\pm y_{J},t\right)=0$ is expressed by the stationary parabolic response across the lane-junction according to Equation (35).

(35) $$\Delta V_{J}\left(x,y,t\right)=\frac{\rho_{J}y_{J}^{2}}{2\;d_{J}}\left(1-\frac{y^{2}}{y_{J}^{2}}\right)\;i_{M}^{tot}\left(x,t\right)$$

For an adhering axon, we choose $d_{J}^{0}=10\;nm$ as the smallest distance reported for a cell membrane on a silicon chip [50]. The half-width of the junction is $y_{J}=0.1\;\mu m$ for $a_{M}=0.5\;\mu m$. With $\rho_{J}=100\;\Omega cm$, the sheet resistance is $r_{J}=100\;M\Omega$. The profile $\Delta V_{J}\left(x,y\right)$ at an arbitrary time is plotted in Figure 11 (bottom). The response along the lane is almost biphasic positive-negative with peak and trough separated by 120 *µm* in space and 140 *µs* in time. The amplitudes in the center of the junction are 1.3 *µV* and −1.9 *µV*.

The total voltage in the cable-substrate junction is obtained by superposing the voltage drop in the junction and the voltage in the surrounding electrolyte. Considering that the current of an adhering cable flows into one half-space, the amplitudes of the voltage in the electrolyte are 12 *µV* and −18 *µV*. The contribution of the electrolyte is by an order of magnitude larger than the contribution of the cable-substrate junction with 1.3 *µV* and −1.9 *µV*.

**Figure 12:**
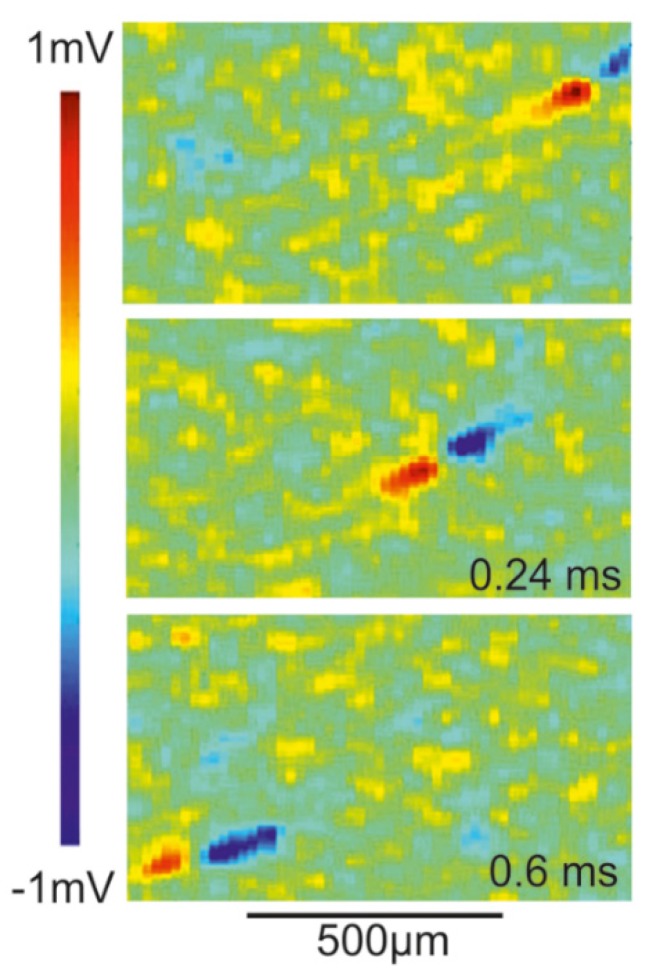
Transistor recording of propagating action potential in a rabbit retina [51]. Color-coded maps of the apparent changes of the gate voltage $\delta V_{Gapp}^{EOMOS}$ recorded with an array of EOMOS transistors at three different times. The action potential propagates from right to left at a velocity of 1.5 *m/s*. The waveform is biphasic positive-negative and represents the profile of a current-induced extracellular voltage with $\delta V_{Gapp}^{EOMOS}=V_{E}$. Peak and trough are separated by 120 *µm* in space corresponding to a separation of 80 *µs* in time.

*Transistor recording of axon in retina*. The extracellular voltage of a propagating AP has been studied by transistor recording of axons in the rabbit retina [51]. The retina was mounted with ganglion cells down on an array of EOMOS transistors. Maps of the transistor response $\delta V_{Gapp}^{EOMOS}$ are displayed in Figure 12. The velocity of the AP is 1.5 *m/s*. The transistor response is biphasic positive-negative with peak and trough separated by 120 *µm* in space and 80 *µs* in time. The amplitudes are about 500 *µV* and −675 *µV*.

The asymmetric biphasic waveform and the separation of peak and trough correspond to the computation shown in Figure 11. The agreement indicates that the transistor response is due to a current-induced response with $\delta V_{Gapp}^{EOMOS}=V_{E}$. The velocity may be matched by choosing a larger radius or lower intracellular resistivity according to the relation $v_{AP}^{}=\sqrt{K\cdot a_{M}/2\rho_{cell}\;c_{M}}$ with $K=10.47\;ms^{-1}$ [30]. Striking is the large amplitude of the recordings. Two issues must be considered: (i) for an axon near the substrate, even without close adhesion, the whole membrane current flows into a half-space with the mounted retina; and (ii) the retina has a resistivity of about 1250 Ω*cm* [52]. Thus, the result of the computation shown in Figure 11 must be multiplied by a factor 25, yielding amplitudes of 150 *µV* and −240 *µV*. The remaining difference with respect to the experiment may due to a larger axon radius or higher membrane conductances.

*Transistor recording with adhering axon in culture*. The extracellular voltage of a propagating AP has been studied for adhering axons of cultured mammalian neurons using an array of metal electrodes on a CMOS chip (see Appendix B) [53]. The amplitudes of the recordings were in the range of 10 *µV*. Peak and trough of biphasic signals were separated by about 150 *µs*. These values are in agreement with the computation shown in Figure 11.

The propagation of an AP along an adhering mammalian axon has also been probed with an array of nanowire transistors on a planar substrate [54]. Almost monophasic positive transients of the nanowire current were reported corresponding to an extracellular voltage in the range of −85 *mV* [55] or −6.6 *mV*, respectively [56]. Even the lower estimate is four orders of magnitude larger than computed for the voltage drop in a cable-substrate junction and three orders of magnitude larger than computed for the voltage in the surrounding electrolyte. It is difficult to see how the parameters could be changed to avoid that discrepancy. Moreover, the waveform of the reported transients, which resemble the inverted intracellular voltage of a local AP, are difficult to reconcile with the waveform of the extracellular voltage of a propagating AP [57].

## 9. Receptor-Cell-Transistor Sensor

Transistor recording with intact cells is an approach to develop biosensors with cellular receptors that bind agonists or pharmaceuticals. The receptor may activate an ion conductance in the plasma membrane, which gives rise to a current-induced response of the transistor. Considering that ligand-activated conductances remain open for a long time due to slow inactivation, a change of the extracellular ion concentrations is expected. An evaluation in terms of a current-induced response requires a gate oxide with a weak ion sensitivity. First, the concept is considered with the one-compartment model for constant ion concentrations (quasi-stationary response, small voltages), and then the prototype with a serotonin receptor is described.

*Receptor-activated conductance.* We assume that the receptor-activated conductance with a high reversal voltage $V_{M0}^{rec}$ dominates the membrane. According to the two-domain model of Equation (29), the current balances in the junction and in the cell are expressed by Equation (36), with a receptor-activated conductance $g_{JM}^{rec}$ in the adherent membrane and an average conductance ${\bar{g}}_{M}^{rec}$.

(36a) $$g_{JM}^{rec}\left(V_{M}-V_{M0}^{rec}\right)+c_{M}\frac{dV_{M}}{dt}=g_{J}V_{J}$$

(36b) $${\bar{g}}_{M}^{rec}\left(V_{M}-V_{M0}^{rec}\right)+c_{M}\frac{dV_{M}}{dt}=0$$

When the conductance is activated, the intracellular voltage relaxes rapidly towards the reversal voltage according to Equation (36b). According to Equation (36a), there is no stationary junction voltage, only a short transient, which is difficult to detect.

*Supplementary K conductance.* A stationary driving voltage $V_{M}-V_{M0}^{rec}$ is established if a voltage-activated potassium conductance $g_{M}^{K}$ is added with $V_{M0}^{K}<V_{M0}^{rec}$. The junction voltage results from a superposition of the ion currents of both conductances according to Equation (37a). According to Equation (37b). The intracellular voltage approaches a resting voltage $V_{M0}^{K}<V_{M}^{R}<V_{M0}^{rec}$.

(37a) $$g_{JM}^{rec}\left(V_{M}^{R}-V_{M0}^{rec}\right)+g_{M}^{K}\left(V_{M}^{R}-V_{M0}^{K}\right)=g_{J}V_{J}$$

(37b) $${\bar{g}}_{M}^{rec}\left(V_{M}^{R}-V_{M0}^{rec}\right)+g_{M}^{K}\left(V_{M}^{R}-V_{M0}^{K}\right)=0$$

If the potassium current is eliminated, we obtain Equation (38), where the junction voltage is determined by the driving voltage $V_{M}^{R}-V_{M0}^{rec}$ and the inhomogeneity of the receptor-activated conductance.

(38) $$\left(g_{JM}^{rec}-{\bar{g}}_{M}^{rec}\right)\left(V_{M}^{R}-V_{M0}^{rec}\right)=g_{J}V_{J}$$

On this basis, the transistor sensorics is described as follows: a cell stays at its low natural resting voltage with a deactivated receptor conductance and a deactivated K conductance. The agonist is added, and current flows into the cell through the receptor-activated conductance. The intracellular voltage rises such that the K conductance is activated. K current flows out of the cell, and a resting voltage $V_{M0}^{K}<V_{M}^{R}<V_{M0}^{rec}$ is established. The superposition of the two ion currents through the adherent membrane yields a junction voltage $V_{J}>0$ for a depletion $g_{JM}^{rec}<{\bar{g}}_{M}^{rec}$ and a junction voltage $V_{J}<0$ for an enhancement $g_{JM}^{rec}>{\bar{g}}_{M}^{rec}$. Finally, the receptor-activated conductance is inactivated, the intracellular voltage drops, the K conductance is deactivated, and the natural resting voltage is reestablished.

*Inhomogeneity induced by diffusion.* It is not necessary that the inhomogeneity of a receptor-activated conductance is structurally implemented. When an agonist is added to the bath, it first reaches the free membrane of an adherent cell and, with a delay, the adherent membrane due to diffusion of the agonist along the cell-substrate junction. The first phase with an activated free membrane is equivalent to $g_{JM}^{rec}<{\bar{g}}_{M}^{rec}$. The second phase with an activated adherent membrane and an inactivated conductance in the free membrane is equivalent to $g_{JM}^{rec}>{\bar{g}}_{M}^{rec}$. Prerequisite of such an effect is a matched dynamics of diffusion and inactivation.

**Figure 13:**
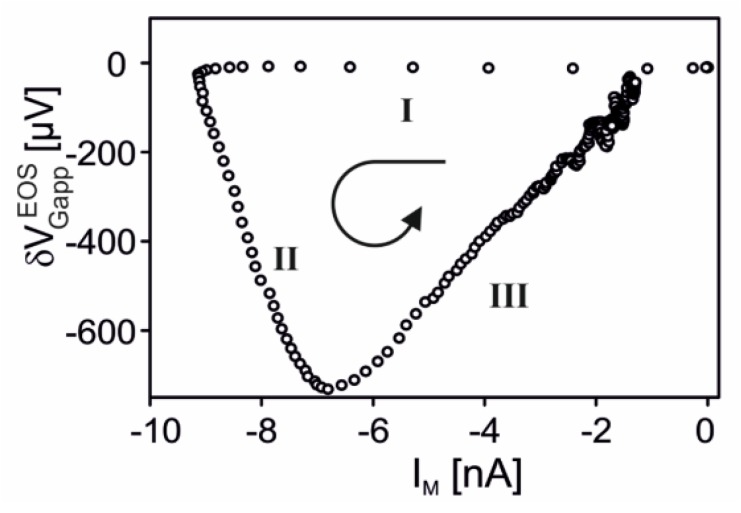
Transistor recording of the serotonin receptor 5-HT3A in a HEK293 cell for constant intracellular voltage (by permission [5]). The apparent change of the gate voltage $\delta V_{Gapp}^{EOS}$ is plotted versus the whole-cell current $I_{M}$ upon fast addition of 100 *µM* serotonin at an intracellular voltage of −120 *mV*. The time direction is indicated by the arrow (data interval 2.5 *ms*). The recording is obtained with a low ion sensitivity of the gate oxide. Three phases are distinguished: (I) increasing whole-cell current without transistor signal (about 20 *ms*); (II) decreasing whole-cell current and increasing transistor signal (about 70 *ms*); and (III) decreasing whole-cell current and decreasing transistor signal.

The delayed activation of the adherent membrane by diffusion has been demonstrated for the serotonin receptor 5-HT3A in a HEK293 cell under voltage-clamp [58]. A transistor with a weak ion sensitivity was used as described above. Serotonin (100 *µM*) was added with a double-barreled pipette. The apparent change of the gate voltage $\delta V_{Gapp}^{EOS}$ is plotted in Figure 13 versus the total membrane current $I_{M}$. Three phases can be distinguished: (i) the membrane inward current increases for 20 *ms* without response of the transistor due to an activation of the free membrane; (ii) the inward current decreases and the apparent change of the gate voltage increases for 70 *ms* due to inactivation of the free membrane and activation of the adherent membrane; and (iii) both the membrane current and the transistor signal disappear due to complete inactivation.

**Figure 14:**
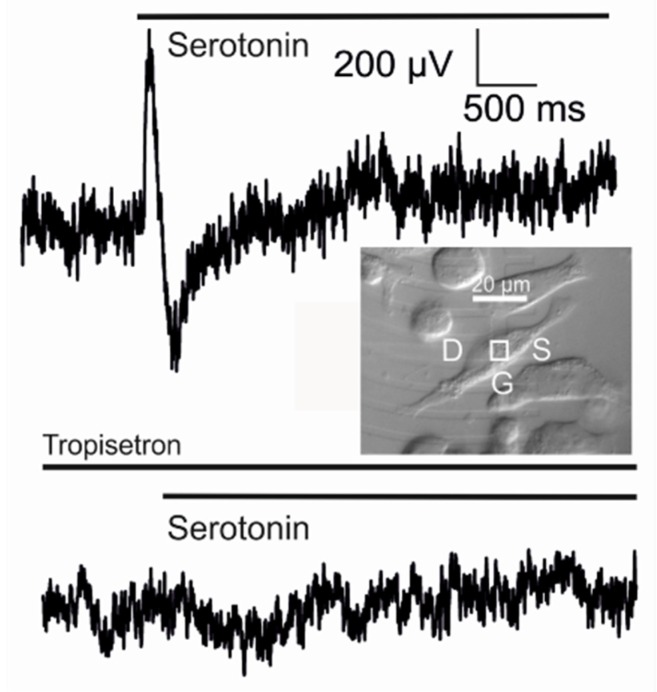
Transistor recording of the serotonin receptor 5-HT3A with the potassium channel Kv1.3 in HEK293 cell [35] for a low ion sensitivity of the gate oxide. The upper trace shows the apparent change of the gate voltage $\delta V_{Gapp}^{EOS}$ upon application of 100 *µM* serotonin. The biphasic transient reflects the current-induced extracellular voltage in the cell-substrate junction. The lower trace is a control experiment with the same cell in the presence of 1 *µM* tropisetron, a selective antagonist of the 5-HT3A receptor. The micrograph shows the position of cell and transistor (source, gate, drain).

*Transistor sensorics with serotonin receptor.* A complete biosensor with the 5-HT3A receptor has been implemented with a supplementary Kv1.3 channel in HEK293 cells on a transistor with a low ion sensitivity of the gate [35]. A result is shown in Figure 14. When serotonin is applied, a positive transistor signal of about 600 *µV* appears. Within about 100 *ms,* it changes to a negative signal of about −450 *µV*, which relaxes slowly. The whole signal is abolished if the specific antagonist tropisetron is added.

The biphasic waveform of the transistor record is expected for a current-induced response with $\delta V_{Gapp}^{EOS}=V_{J}$. The two phases correspond to a switched inhomogeneity of the membrane from depletion to enhancement of the receptor-activated conductance in the junction, as caused by diffusion and inactivation. The asymmetry of the positive and negative wave and the small amplitudes are due to the strong overlap of the two phases as the adherent membrane becomes activated at a time when the free membrane is only partially inactivated.

## 10. Conclusions

Current-induced transistor signals with electrogenic cells in a cell-substrate junction are well understood in terms of a planar capacitive core-coat conductor as it is documented by a wide range of experiments. Apparent changes of the gate voltage are caused by the current-induced extracellular voltage in the junction beyond the electrical double layer of the gate oxide. The signals are determined by the structure of the cell-substrate junction (area, cleft width, resistivity) and by the ion conductances of the cell with an inhomogeneous distribution in the adherent and free membrane. Interface-induced signals due to changing ion concentration are important for longer activations of voltage-gated conductances under voltage-clamp. They play a minor role for transient activations of ion conductances as with firing neurons and can be avoided if the ion sensitivity of the gate oxide is suppressed.

Three issues should be considered with respect to a development of a reliable current-induced transistor sensorics: (i) The inhomogeneous distribution of ion conductances is important. A distinct inhomogeneity of ion conductances in the adherent membrane is documented with recombinant ion channels in HEK293 cells, with cultured mammalian neurons, and with dissociated invertebrate neurons. However, these effects were given without control. The cellular mechanisms for the localization of membrane proteins must be taken into account and applied for the development of designed cell-substrate junctions; (ii) The passivation of the gate oxide with respect to ion binding is important. Chemical processes must be developed that block the interaction of the gate with ions, a goal that is opposite to the goal of classical ISFET sensorics; (iii) The position of recording transistors with respect to the adhering cells is important. Accidental positions and manual positioning with a pipette are not adequate. Controlled local adhesion with a chemically modified surfaces must be considered for optimal recordings.

## Abbreviations

The following abbreviations are used in this manuscript:

CMOS: complementary metal oxide semiconductor
EOMOS: electrolyte oxide metal oxide semiconductor
EOSFET: electrolyte oxide semiconductor field*–*effect transistor
FLIC: fluorescence interference contrast
HEK239: human embryonic kidney
ISFET: ion sensitive field-effect transistor
MOSFET: metal oxide semiconductor field-effect transistor

## Appendix A. Flatband Voltage of EOSFET

The flatband voltage of an EOSFET can be described in analogy to a MOSFET by introducing workfunctions (for the semiconductor and the redox solution of the reference electrode) and surface dipole voltages [6]. The workfunctions are defined as differences of the electrochemical potential of electrons in the vacuum (just beyond the atomic forces) and in the material with $\Phi_{k}=\left(\mu_{}^{el,vac}-\mu_{k}^{el}\right)-e_{0}\left(\varphi_{k}^{vac}-\varphi_{k}^{\infty }\right)$, and the surface dipole potentials as negative differences of the electrical potential across the free surfaces with $-\chi_{i}=\varphi_{i}^{vac}-\varphi_{i}^{S}$. We eliminate the difference $\mu_{red/ox}^{el}-\mu_{Si}^{el}$ in Equation (1), use the approximations $\chi_{red/ox}\approx \chi_{E}$ and $\varphi_{k}^{S}\approx \varphi_{k}^{\infty }$, express the difference $\varphi_{E}^{\infty }-\varphi_{Si}^{\infty }$ according to Equation (2), and introduce the differences of the electrical potential across the quasi*-*Helmholtz layer, across the Gouy-Chapman layer, and in the free electrolyte. For $\Delta \varphi_{Si}^{S}=0$ and $\Delta \varphi_{E}=0$ the flatband voltage of a p*-*channel transistor is given by Equation (A1).

(A1) $$\begin{array}{ll} V_{FB}^{EOS} & =\left(\Phi_{red/ox}-\Phi_{Si}\right)/e_{0}\\ & \;-\left[\Delta \varphi_{GCh}^{}+\Delta \varphi_{Hh}^{}-\left(\chi_{Ox}-\chi_{E}\right)\right]-\sigma_{OxSi}/c_{Ox}+\left[\Delta \varphi_{OxSi}-\left(\chi_{Ox}-\chi_{Si}\right)\right] \end{array}$$

The two brackets account for changes of the difference of the electrical potential between oxide and electrolyte and between oxide and semiconductor, respectively, when the interfaces are formed from the free surfaces. (Formally they describe a difference of the electrical potential in the vacuum as it can be seen when the transfer of a probe charge across the interface is identified with an indirect transfer through the two free surfaces).

Unfortunately, Equation (A1) cannot be used to estimate the flatband voltage in analogy to a MOSFET. There, effective workfunctions from the bulk gate metal and from the bulk semiconductor, respectively, across the interface to the vacuum of the gate oxide can be measured by a photoelectronic approach. For an EOSFET, the workfunction of the redox solution can be obtained from the redox potential [5,6]. However, the jump of the electrical potential across the oxide/electrolyte interface is unknown. It may be fairly large due to the strong hydration of the polar surface [59]. The flatband voltage of the EOSFET must be measured.

## Appendix B. EOSFET and other Sensors

The concept of the current-induced transistor sensorics of the EOSFET with an exposed gate oxide can be applied for other potentiometric sensors, which are used for electrogenic cells to record voltage changes with respect to a reference electrode: (i) With EOMOS transistors on a CMOS chip [60], a thin metal gate is intercalated between the oxide exposed to the electrolyte and the genuine gate oxide. It is used to adjust the working point in calibration phases, but is free floating in recording phases. Every change of the electrical potential in the free electrolyte and at the oxide/electrolyte interface has the same effect as with an EOSFET; (ii) With metal electrodes on a CMOS chip [53] the exposed electrodes are in close contact (and electronically equilibrated) with the gate of a MOSFET. If a polarizable metal is used (*i.e.*, without electronic equilibration with the electrolyte), a changing electrical potential in the free electrolyte or at the metal/electrolyte interface is projected onto the electrical potential at the surface of the gate oxide. Of course, the interface-induced signal differs from an EOSFET due to the different chemistry of the metal surface; (iii) Traditional planar electrodes [61] are electronically equilibrated with a metal wire that leads to the amplifier. For a polarizable electrode, a changing electrical potential in the electrolyte or at the interface is projected onto the wire. Perturbations of amplitude and waveform may occur due to the large separation of electrode and amplifier, and it is advisable to apply suitable calibration voltages to the reference electrode. A certain disadvantage of all arrays with uncoated electrodes is the chemically heterogeneous surface of the substrate, which can affect tissues and cultured cells.

## Appendix C. Capacitive Core–Coat Conductor and Poisson–Nernst–Planck Approach

A cell-substrate junction has a dielectrically inhomogeneous structure with a profile of the dielectric constant ε(z) from semiconductor to oxide to electrolyte to membrane to cytoplasm. With a permittivity $\varepsilon \;\varepsilon_{0}$, and an electrical potential φ, the relation between the electrical displacement and the space charge density $\rho^{ch}$ is expressed by Equation (C1).

(C1) $$-\nabla \left(\varepsilon \;\varepsilon_{0}\;\nabla \varphi \right)=\rho^{ch}$$

A short exercise shows that the difference of the electrical potential across an insulator between two electroneutral bulk electrolytes is determined by thin opposite space charge layers as expressed by a capacitor relation. This result is the basis for the physics of biomembranes. It is applicable for a cell-substrate junction with its two insulating coats if the electrolyte film is distinctly wider than a Debye length of the electrolyte: a cell-substrate junction is a planar capacitive core-coat conductor in analogy to the linear capacitive core-coat conductor of a neuronal cable [9,10,11,12,13]. The theory with changing ion concentrations is described by a drift-diffusion equation (Equation (6)) for the balance of the ion flux and by a capacitor relation (Equation (7)) between the electrical charge and the electrical potential.

In a Poisson-Nernst-Planck approach for a cell–substrate junction [16], the drift-diffusion (Nernst-Planck) equation is the same as for a capacitive core–coat conductor. However, the electrical charge is assigned to the electrolyte film with a space charge density $\rho_{E}^{ch}$, which determines the curvature of the electrical potential along the junction according to the (Poisson) Equation (C2).

(C2) $$-\varepsilon_{E}\;\varepsilon_{0}\nabla^{2}\varphi_{E}=\rho_{E}^{ch}$$

The Poisson-Nernst-Planck approach disregards the capacitive coats of the cell-substrate junction. It describes the slice of an infinite homogeneous volume conductor. So it is certainly incomplete. As a matter of fact, the current-induced voltage in a cell-substrate junction was computed in terms of the capacitive core-coat conductor using the one-compartment (circuit) model for constant ion concentrations (Equation (1) of [16]). In a separate step, the changing ion concentrations were computed with the Nernst-Planck and the Poisson equation (Equations (A1)–(A5) of [16]). The effect of the changing ion concentrations on the current-induced response was not taken into account. This separate computation of the voltage and the concentrations is arbitrary and without any foundation. Moreover, it is also inconsistent: in the circuit model, the charge is proportional to the voltage, and the electrical time constant is in the microsecond range, whereas in the Poisson-Nernst-Planck approach, the charge is proportional to the curvature of the voltage profile, and the electrical time constant is in the nanosecond range.

Despite its deficiencies, the approach yielded results (for the voltage-clamp situation) that are similar (though not identical) to those obtained with the capacitive core-coat conductor for two reasons: (i) the missing concentration dependence of the current-induced voltage (trace 1 in Figure 5e) can be compensated by the interface-induced response with an appropriate ion sensitivity of the gate oxide (trace 3 in Figure 5e); and (ii) the Poisson equation, which substitutes the curvature of the voltage profile by the electrical charge has a similar effect as the formal substitution $-\nabla^{2}\Rightarrow \eta_{J}/A_{J}$ used to derive the one-compartment model. A relation for the drift component of the concentration dynamics is obtained from Equations (23) and (24b) that is analogous to Equation (A4) of [16] when the permittivity of the core electrolyte is replaced by the capacitance of the coat as $\varepsilon_{E}\varepsilon_{0}\Rightarrow c_{J}A_{J}/d_{J}\eta_{J}$. The time constant is in the range of 1 *µs* instead of 0.1 *ns*, a difference that is irrelevant when the quasi–stationary state is evaluated. Thus the approach of [16] may be considered as a rough approximation of the capacitive core-coat conductor.
